# Social status shapes the bacterial and fungal gut communities of the honey bee

**DOI:** 10.1038/s41598-018-19860-7

**Published:** 2018-01-31

**Authors:** Ji-Hyun Yun, Mi-Ja Jung, Pil Soo Kim, Jin-Woo Bae

**Affiliations:** Department of Life and Nanopharmaceutical Sciences and Department of Biology, Kyung Hee University, Seoul, 130-701 Korea

## Abstract

Despite the fungal abundance in honey and bee bread, little is known about the fungal gut community of the honey bee and its effect on host fitness. Using pyrosequencing of the *16S rRNA* gene and ITS2 region amplicons, we analysed the bacterial and fungal gut communities of the honey bee as affected by the host social status. Both communities were significantly affected by the host social status. The bacterial gut community was similar to those characterised in previous studies. The fungal gut communities of most worker bees were highly dominated by *Saccharomyces* but foraging bees and queens were colonised by diverse fungal species and *Zygosaccharomyces*, respectively. The high fungal density and positive correlation between *Saccharomyces* species and *Lactobacillus* species, known yeast antagonists, were only observed in the nurse bee; this suggested that the conflict between *Saccharomyces* and *Lactobacillus* was compromised by the metabolism of the host and/or other gut microbes. PICRUSt analysis revealed significant differences in enriched gene clusters of the bacterial gut communities of the nurse and foraging bees, suggesting that different host social status might induce changes in the gut microbiota, and, that consequently, gut microbial community shifts to adapt to the gut environment.

## Introduction

The honey bee, *Apis mellifera*, is a social insect, since social status depends on their role within the colony. Social status corresponds to the worker development stage. Younger bees (namely, nurse bees) look after their broods and the queen, repair and construct the comb, and evaporate the nectar to produce honey, whereas older bees (namely, foraging bees) gather resources such as nectar, pollen, propolis, and water^1^.

In recent decades, a significant decline of honey bee colonies has been observed worldwide^2,3^. Honey bee decline could not only lead to world economic losses but also be associated with an immense global health burden: a modelling analysis^4^ indicated that pollinator loss causes a shortage of pollinator-dependent crops and their replacement with other crops may have implications for human health. Specifically, the sudden loss of adult worker bee has been named Colony Collapse Disorder (CCD)^5^. The forces predicted to drive the CCD are pathogen-associated stress^6–8^, habitat loss^9^, and pesticides^10^. Other recent studies suggested that the CCD may be caused by a combination of multiple factors^3,8^. CCD-affected colonies are characterised by higher pathogen loads and other co-infecting pathogens than control colonies, suggesting either a higher pathogen exposure or reduced defence response of the CCD-affected bees^5^. A previous study shows that alterations in the gut microbiota induced by dysbiosis of the host innate immune system eventually lead to host mortality^11^. These observations have led researchers to pay attention to interactions between honey bees and their gut microbes and pathogens^12,13^.

Previous studies reported that the bacterial gut community of the *Apis mellifera* is comprised by eight to nine taxa^14–16^: ‘Alpha-1’ (*Bartonella apis*)^17^, ‘Alpha-2.1’ (Acetobacteraceae), ‘Alpha-2.2’ (“*Parasaccharibacter*” or *Bombella*)^18,19^, ‘Beta’ (*Snodgrassella alvi*)^20^, ‘Bifido’ (*Bifidobacterium asteroides* and *Bifidobacterium coryneform*)^21^, ‘Firm-4 and Firm-5’ (*Lactobacillus* sp.)^22^, ‘Gamma-1’ (*Gilliamella apicola*)^20^ and ‘Gamma-2’ (*Frischella perrara*)^23^. Genomic analysis and *in vitro* assays revealed that members of the gut microbiota play important roles in enhancing the life quality of the host. The genes encoding pectin-degrading enzymes are highly abundant in the gut metagenome of the honey bee and *Gilliamella* isolates represented the pectinase activities suggesting that *Gilliamella* contribute to pectin degradation^24^. Biofilm formation-related genes are enriched in *S*. *alvi* and *G*. *apicola* of the honey bee^24^, and fluorescence microscopy revealed that the epithelium layer of the host ileum is enveloped by the two symbionts^25^, suggesting that the biofilm functions as a protective layer against parasite invasion. *In vitro* assays showed that lactic acid bacteria and *Parasaccharibacter apium* from the honey bee gut protect the host from the American foulbrood causative agents *P*. *larvae* and *Nosema*^12,26^, respectively. Colonisation with *F*. *perrera* triggers scab formation, expected to a melanisation response, in the honey bee pylorus, suggesting activation of the insect immune system^27^.

The role of symbiotic fungi as a food source for Hymenoptera order has been demonstrated. For example, in the association between the fungus *Monascus* sp. and the Brazilian stingless bee, *Scaptotrigona depilis*, the bee cultivates the fungus inside the brood cells^28^; further, leaf cutter ants (two Genera *Acromyrmex* and *Atta*) also cultivate the fungus as the food source in their garden, which is pathogen-controlled by symbiotic bacteria^29^. The bacterial and fungal interactions in the gut of the *Megachile* bee, which may be key players for regulation of the gut microbial community, were proposed in a recent study^30^. Research on the nosemosis field tests revealed that the degree of infection with *Nosema ceranae* is associated with yeast proliferation in the honey bee gut, suggesting that exposure to such stresses as infection may result in structural changes of the gut microbial community^31^.

Although the symbiotic relationships between hymenopteran and fungi have been reported^32^ and fungi are abundant in the bee bread and nectar^33,34^, little is known about the fungal community of the honey bee and its effect on host fitness. Gilliam *et al*. reported four fungal species isolated from healthy honey bees, but not yeasts^35^ and *Candida* species are predominantly isolated from honey bee fed herbicide and antibiotics^36^. Whereas sequencing the gut metagenome of the honey bee revealed the existence of *Saccharomycetaceae* such as *Saccharomyces* and *Zygosaccharomyces*^37^, sequencing the metatranscriptome revealed that the honey bee gut contains a small fraction of microbial eukaryotic transcripts^38^, suggesting that different studies may obtain different results.

In the current study, we investigated the shift in gut microbiota of the adult honey bee, *Apis mellifera*, depending on the host social status, using 454 amplicon assays of the *16S rRNA* gene and Internal transcribed spacer 2 (ITS2) region. We predicted the function of gut bacteria using the PICRUSt (Phylogenetic Investigation of Communities by Reconstruction of Unobserved States) programme, and quantified copy numbers of the *16S rRNA* gene and ITS2 region. Finally, we explored the link between the fungal and bacterial community of the honey bee according to the social status of the host.

## Results

### Analysis of pyrosequencing data

In total, 34,980 high-quality bacterial sequences and 107,224 high-quality fungal sequences were obtained, with 636 bacterial operational taxonomic units (OTUs) and 895 fungal OTUs identified at the 97% sequence similarity cut-off. The average read length was 332.52 bp for bacteria and 408.36 bp for fungi, and each individual sample was covered by an average of 744.26 (±78.75) and 2,749.33 (±702.40) of bacterial and fungal reads, respectively. The average number per bee of bacterial OTUs was 48.77 (±3.52); for fungal OTUs it was 62.38 (±10.15) (Table S1). Good’s coverage, estimating the OTU% in the honey bee samples, averaged 0.96 (±0.004) for bacteria and 0.96 (±0.025) for fungi, suggesting that the obtained values could represent the overall structure and composition of the honey bee gut microbiota.

### Comparison of diversity indices of bacterial and fungal communities

**Figure 1:**
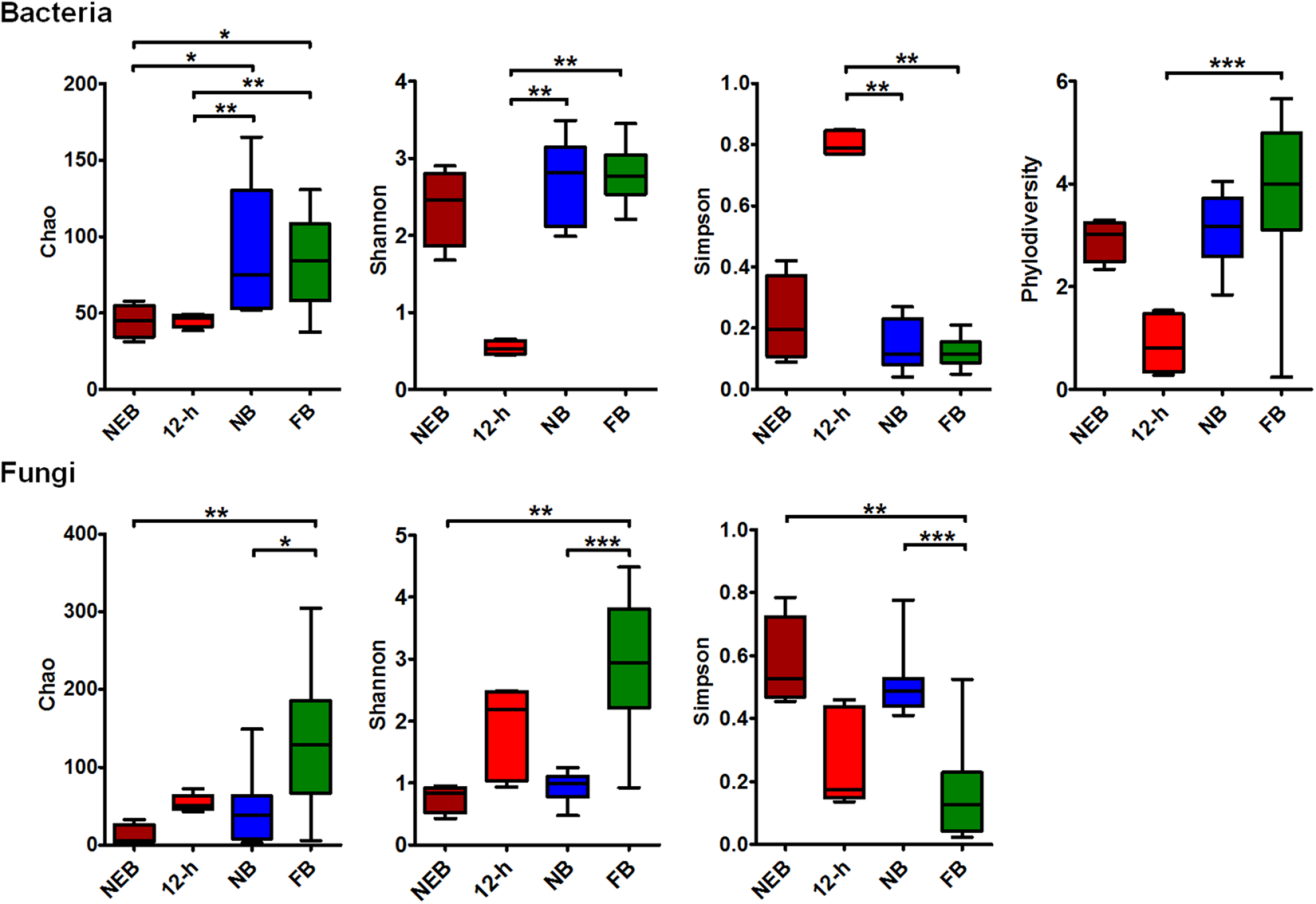
Comparison of alpha-diversity indices of the gut microbial community in honey bees performing four social roles. Box plots depict the medians (central horizontal lines), inter-quartile ranges (boxes), and 95% confidence intervals (whiskers). NEB, newly-emerged bee; 12-h, 12-h-old bee; NB, nurse bee; FB, foraging bee. An FDR adjusted p-value are from Kruskal-Wallis test. Asterisks indicate statistically significant differences between pairs of values (*P < 0.05, **P < 0.01, ***P < 0.001, and ****P < 0.0001).

OTU richness and diversity were calculated with the phylodiversity, Chao, Ace, Jackknife, Shannon, and Simpson parameters using the mothur programme. Both the average numbers of the observed and estimated fungal OTUs were higher than the bacterial numbers (Supplementary Table S1). Microbial richness and diversity in the gut were affected by the host social status (Fig. 1), and significant differences were observed in the composition and structure of microbial OTUs between hosts with different social status. Namely, for bacteria, the *P*-values for significance of the diversity indices were as follows: Ace, *P* = 0.0213; Chao, *P* = 0.0012; Shannon, *P* = 0.0021; Simpson, *P* = 0.0018; phylodiversity, *P* = 0.0013. For fungi, these were: Ace, *P* = 0.0018; Chao, *P* = 0.0014; Shannon, *P* < 0.0001; Simpson, *P* = 0.0001. The bacterial and fungal OTU indices were lowest in the queen and in the newly-emerged bee (Supplementary Table S1), respectively, whereas those of the foraging bee were the highest, implying that a significant increase in OTU numbers is associated with the honey bee social status rather than host aging.

### Microbial community dynamics as affected by the social status of the host

Although eight bacterial phyla were detected in total, the majority of sequences belonged to Firmicutes (56.65%) and Proteobacteria (42.16%), which were previously shown to constitute the majority of the honey bee gut-associated phylotypes^15^. The other phyla detected were Bacteroidetes (0.55%), Actinobacteria (0.41%), Cyanobacteria (0.14%), Tenericutes (0.04%), Fusobacteria (0.04%) and Acidobacteria (0.006%) (Fig. S1a). The dominant bacterial phylum compositions were significantly affected by the social status of the honey bee (Supplementary Fig. S1b).

**Figure 2:**
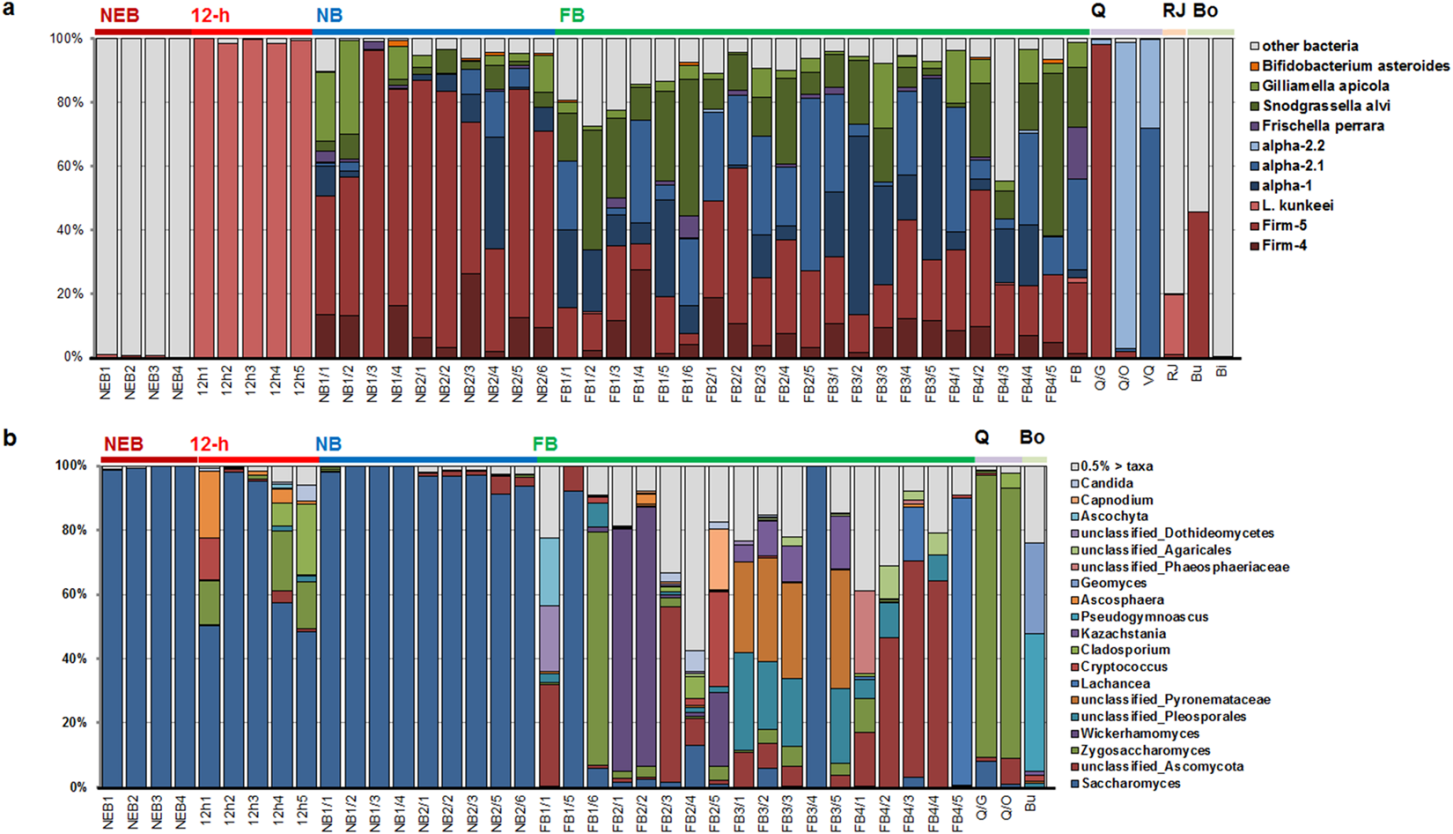
Composition of the gut microbial community. A shift in the gut microbial community, as affected by the honey bee social status, is represented (**a)** at the resident bacterial taxa and (**b**) at the major fungal genus level (>0.5% of all sequences). NEB, newly-emerged bee; 12-h, 12-h-old bee; NB, nurse bee; FB, foraging bee; Q, queen; RJ, royal jelly; Bo, *Bombus*.

Proteobacteria, including Enterobacteriaceae, Sphingomonadaceae, Moraxellaceae and Comamonadaceae dominated the gut of the newly-emerged bee. However, after 12 h, the predominant gut microbiota changed from Proteobacteria to Firmicutes, mainly *Lactobacillus kunkeei* (Fig. 2a and Supplementary Fig. S1b). Nine taxa that are known as conserved intestinal microbes of the honey bee appeared at the nurse bee phase (Fig. 2a). Gut bacterial communities of the nurse bee and the foraging bee were similar in structure, but the relative abundances of the dominant taxa were different depending on the host social status. The relative abundance of ‘Firm-5’ (belonging to Firmicutes) was significantly higher in nurse bees than in foraging bees (*P* = 0.0064). ‘Alpha-2.1’ and ‘*Snodgrassella*’, belonging to Proteobacteria, were significantly more abundant in foraging bees than in nurse bees (*P* = 0.0245; *P* = 0.005) (Fig. 2a and Supplementary Table S2). Non-resident gut bacteria were more abundant in the foraging bee than in the nurse bee (*P* = 0.0445), suggesting that the foraging activity allows the foraging bee to ingest the diverse microbes. The gut of the non-reproductive queen was dominated by ‘Alpha-2.1’ and ‘Alpha-2.2’, whereas the gut and ovary of the naturally-mated queen were dominated by ‘Firm-5’ and ‘Alpha-2.2’, respectively (Fig. 2a).

In total, five fungal phyla were identified within the honey bee intestine. Ascomycota (93.28% of the classified sequences) and Basidiomycota (5.89%) were detected as the dominant phyla, followed by Glomeromycota (0.24%), Chytridiomycota (0.20%), and Zygomycota (0.05%), and 373 fungal sequences (0.35%) were not assigned using the UNITE database (Supplementary Fig. S1a). Regarding the fungal composition, most individual bees were dominated at the phylum level by Ascomycota (Supplementary Fig. S1c). Other phyla (i.e., Basidiomycota, Chytridiomycota and Glomeromycota) appeared at the foraging bee stage. Samples from the queen were also dominated by Ascomycota.

At the genus level, the newly-emerged bee, 12-h-old bee, and nurse bee were dominated by *Saccharomyces*, with proportions of 99.53 ± 0.31%, 69.93 ± 11.05% and 97.06 ± 1.00%, respectively. On the other hand, the foraging bee gut had a low composition ratio of *Saccharomyces* (2.18 ± 0.88%) and comprised multiple fungal taxa (Fig. 2b). The relative abundance of Basidiomycota such as Coprinopsis, Schizopora, and unclassified Agaricales and unclassified Pleosporales belonging to Ascomycota was significantly higher in foraging bees than in nurse bees (Supplementary Table S2). The relative abundance of *Cystofilobasidium* was significantly higher in nurse bees than in foraging bees, and there was a significant difference in *Yarrowia* in 12-h-old bees (Supplementary Table S2) than in other bee group. The gut and ovary of the queen were dominated by Z*ygosaccharomyces* (86.04%) rather than *Saccharomyces* (4.40%).

### Beta diversity analysis and predictive functional analysis of the gut bacteria and fungi

Interaction network analyses using Cytoscape revealed that the host nodes were more connected within the same social status than between honey bee hosts with different status. This indicated that the microbial gut communities of the same social status hosts were more similar to each other than to those from different social status hosts (Supplementary Fig. S2).

**Figure 3:**
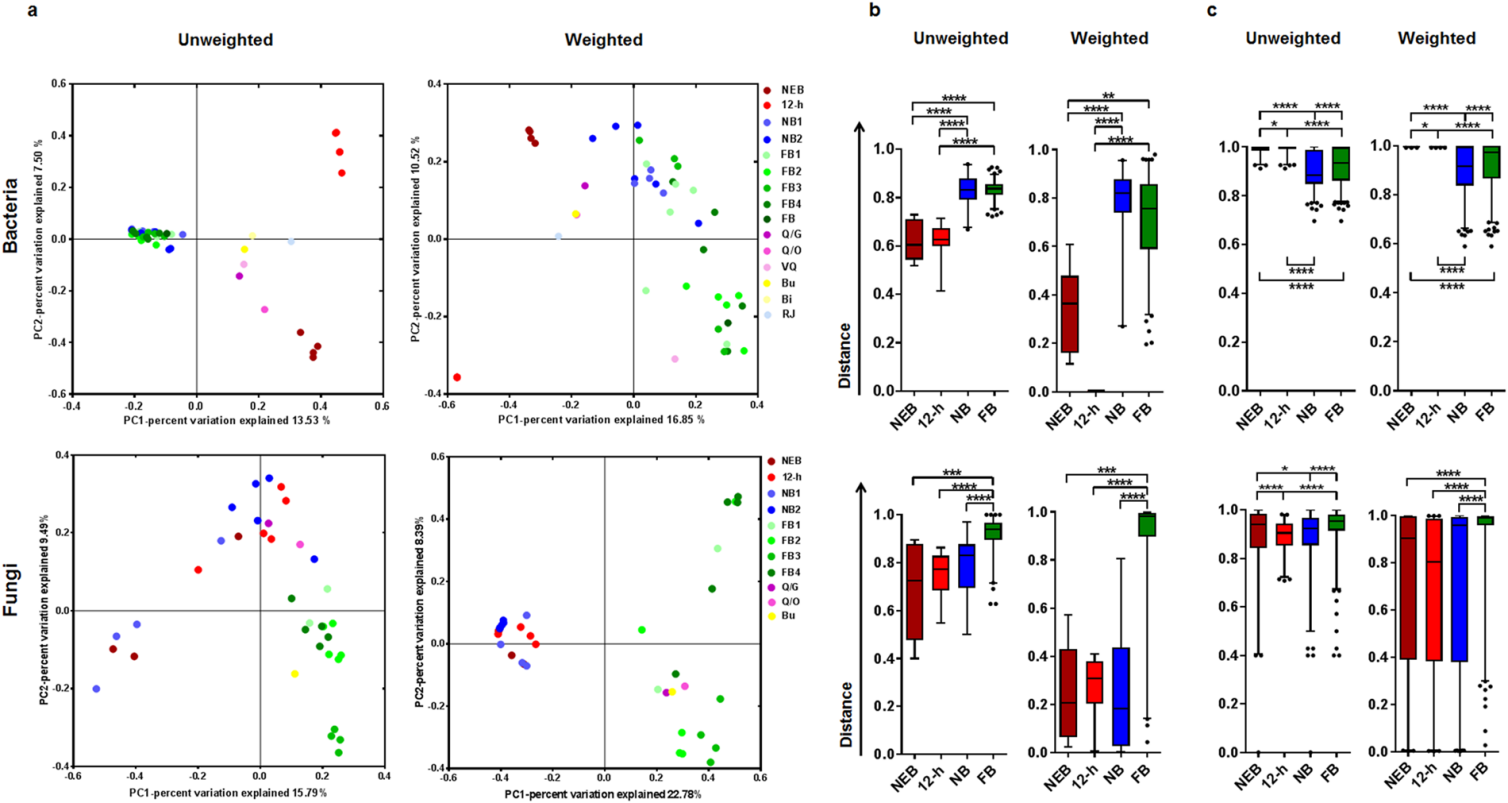
Changes in the gut microbial community depending on the honey bee social status. (**a**) The honey bee gut bacterial and fungal communities clustered using PCoA of the unweighted Jaccard-based and weighted thetaYC-based matrices. Group names are designated by initials, with different colours representing categories, described in Table S1. Nonparametric ANOVA tests were used to test (**b**) intra-group dissimilarity and (**c**) inter-group dissimilarity based on the unweighted Jaccard- and weighted thetaYC-based distances. Box plots depict medians (central horizontal lines), the inter-quartile ranges (boxes), 95% confidence intervals (whiskers), and outliers (black dots). Upper panel, bacteria; lower panel, fungi. NEB, newly-emerged bee; 12-h, 12-h-old bee; NB, nurse bee; FB, foraging bee. An FDR adjusted *p*-value are from Kruskal-Wallis test. Asterisks indicate statistically significant differences between pairs of values (*P < 0.05, **P < 0.01, ***P < 0.001, and ****P < 0.0001).

The principal coordinate analysis (PCoA) and non-metric multidimensional scaling (NMDS) based on the Jaccard matrix (unweighted) or ThetaYC matrix (weighted) analyses revealed that the composition of the gut microbiota clustered according to the host social status (Fig. 3a and Supplementary Fig. S3). The principal coordinate 1 (PC1) of PCoA for the bacterial community separated the newly-emerged bee and the 12-h-old bee from the nurse bee and the foraging bee (Fig. 3a). Although the stress values from NMDS ordination were high (Jaccard: stress = 0.389, R = 0.37; thetaYC: stress = 0.341, R = 0.45), each plot was separated according to the social status. Axis 2 of unweighted NMDS and axis 1 of weighted NMDS separated the foraging bee from the nurse bee (Supplementary Fig. S3). In fungal analysis, PC1 of PCoA, axis 2 of unweighted NMDS (Jaccard: stress = 0.364; R = 0.436), and axis 1 of weighted NMDS (thetaYC: stress = 0.262; R = 0.700) separated the foraging bee from the nurse bee (Fig. 3a and Supplementary Fig. S3). AMOVA and ANOSIM (Bonferroni’s post-hoc test) based on the Jaccard or ThetaYC matrix revealed significant differences in the bacterial gut community of honey bees, which were dependent on their social status (Jaccard: AMOVA, *Fs* = 2.759, *P* = 0.0014; Jaccard: ANOSIM, *r* = 0.817, *P* < 0.001; ThetaYC: AMOVA, *Fs* = 4.810, *P* = 0.0014; ThetaYC: ANOSIM, *r* = 0.831, *P* = 0.001). The fungal gut community of the honey bee also showed significant differences according to social status (Jaccard: AMOVA, *Fs* = 1.842, *P* < 0.001; Jaccard: ANOSIM, *r* = 0.183; *P* = 0.02; ThetaYC: AMOVA, *Fs* = 4.187, *P* < 0.001; ThetaYC: ANOSIM, *r* = 0.234, *P* = 0.01). Statistical analysis using Jaccard or ThetaYC matrix-based distances revealed that inter-individual differences within foraging and nurse bees (excluding the low inter-individual distance of the fungal community of nurse bees in the weighted-based matrix) were significantly higher than those between other groups; however, the intra-group differences were much smaller than the inter-group differences (Fig. 3b and c). Consequently, these results supported the existence of an association between the microbial community members and the host social status, and provided an independent validation of the social status clustering observed in network diagrams. Clustering with respect to sampling site was observed only in unweighted PCoA analyses of the fungal community in foraging bees (supplementary figure S4).

To identify specific taxa in the gut microbiota that were affected by the social status of the host, we performed linear discriminant analysis effect size (LEfSe) analysis (Supplementary Figs S5–6). This analysis revealed that changes in the abundance of 59 bacterial and 62 fungal taxa accounted for the observed differences in the gut microbiota associated with the host social status, suggesting a correlation between those roles and specific subsets of gut microbes. Similarly to statistical analysis using ANOVA (Supplementary Table S2), the relative abundances of *Lactobacillus* Firm-5 (LDA = 5.4975 and *P* < 0.0001) in the nurse bee, Acetobacteraceae alpha-2.1 (LDA = 4.9789 and *P* < 0.0001) and *Snodgrassella* (LDA = 4.9832 and *P* < 0.0001) in the foraging bee, and *L*. *kunkeei* (LDA = 5.7044 and *P* < 0.0001) in the 12-h-old bee, were significantly higher than those in other worker groups. The relative abundances of *Saccharomyces* (LDA = 4.9065 and *P* < 0.0001) in newly-emerged bee, *Zygosaccharomyces* (LDA = 3.8803 and *P* = 0.0007), *Ascosphaera* (LDA = 3.7165 and *P* = 0.0353) and *Candida* (LDA = 3.0292 and *P* = 0.013) in the 12-h-old bee, unclassified Ascomycota (LDA = 4.2546 and *P* = 0.0006), unclassified Pleosporales (LDA = 3.9205 and *P* = 0.0003), *Wickerhamomyces* (LDA = 3.8109 and *P* = 0.0256), unclassified Agaricales (LDA = 3.1296 and *P* < 0.0001) and unclassified Dothideomycetes (LDA = 3.1204 and *P* = 0.0159) in the foraging bee, were significantly higher than those other groups. Statistical analysis using ANOVA also revealed a significant difference in relative abundance of unclassified Agaricales and unclassified Pleosporales in foraging bees (Supplementary Table S2).

**Figure 4:**
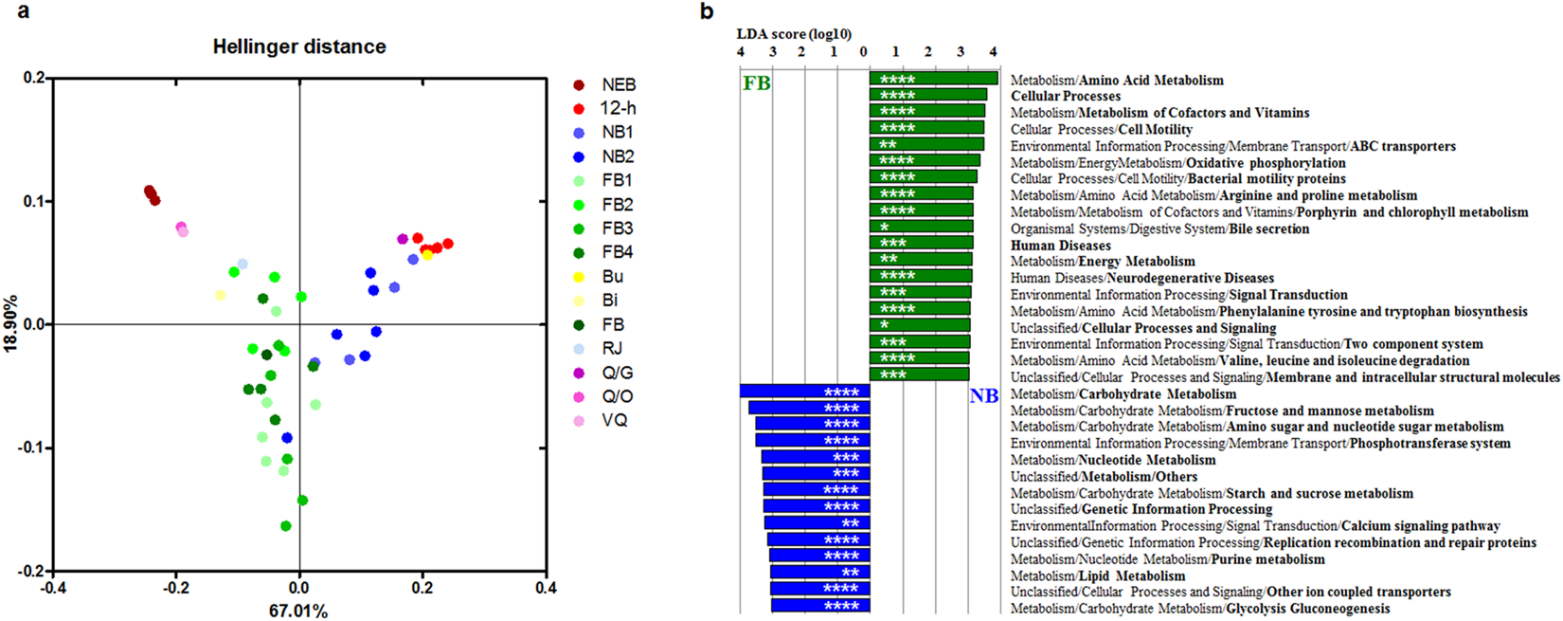
Comparison of the predictive functions of bacterial communities using *16S rRNA* marker gene sequences. (**a**) PCA representing changes in the total gene family at the KEGG level 3 of bacterial communities, according to the honey bee social roles. (**b**) Comparison of the profiles of differentially enriched genes between the nurse bee and foraging bee. The threshold of the LDA score was 3.0. NEB, newly-emerged bee; 12-h, 12-h-old bee; NB, nurse bee; FB, foraging bee. *P*-values are carried out with unpaired t-test with Welch’s correction. Asterisks indicate statistically significant differences between the pairs of values (*P < 0.05, **P < 0.01, ***P < 0.001, and ****P < 0.0001).

Next, to test whether the changes in gut microbial taxa would alter the gut microbiota function, we predicted the functional gene content of bacterial communities by PICRUSt analysis. The analysis of level-3 Kyoto Encyclopedia of Genes and Genomes (KEGG) functional classes revealed a significant difference in functional gene categories between the honey bee groups. To analyse the most pronounced functional differences between the honey bee groups, we performed the Principal Composition Analysis (PCA) using relative abundances of gene family in each sample. Hellinger distance-based PCA revealed that the bacterial gene profile clustered according to the host social status (Fig. 4a). The PCA of gene family abundances revealed that functional gene clusters of the nurse bee and foraging bee were more similar than those of the other worker groups. Genetic differences between the nurse bee and foraging bee gut microbiota were identified by LEfSe based on the linear discriminant analysis (LDA) score 3.0, and significant differences in various carbohydrate metabolism- (*P* = 0.00001) and amino acid metabolism-related genes (*P* = 0.0002) were detected (Fig. 4b).

### The relationship between the bacterial and fungal communities

**Figure 5:**
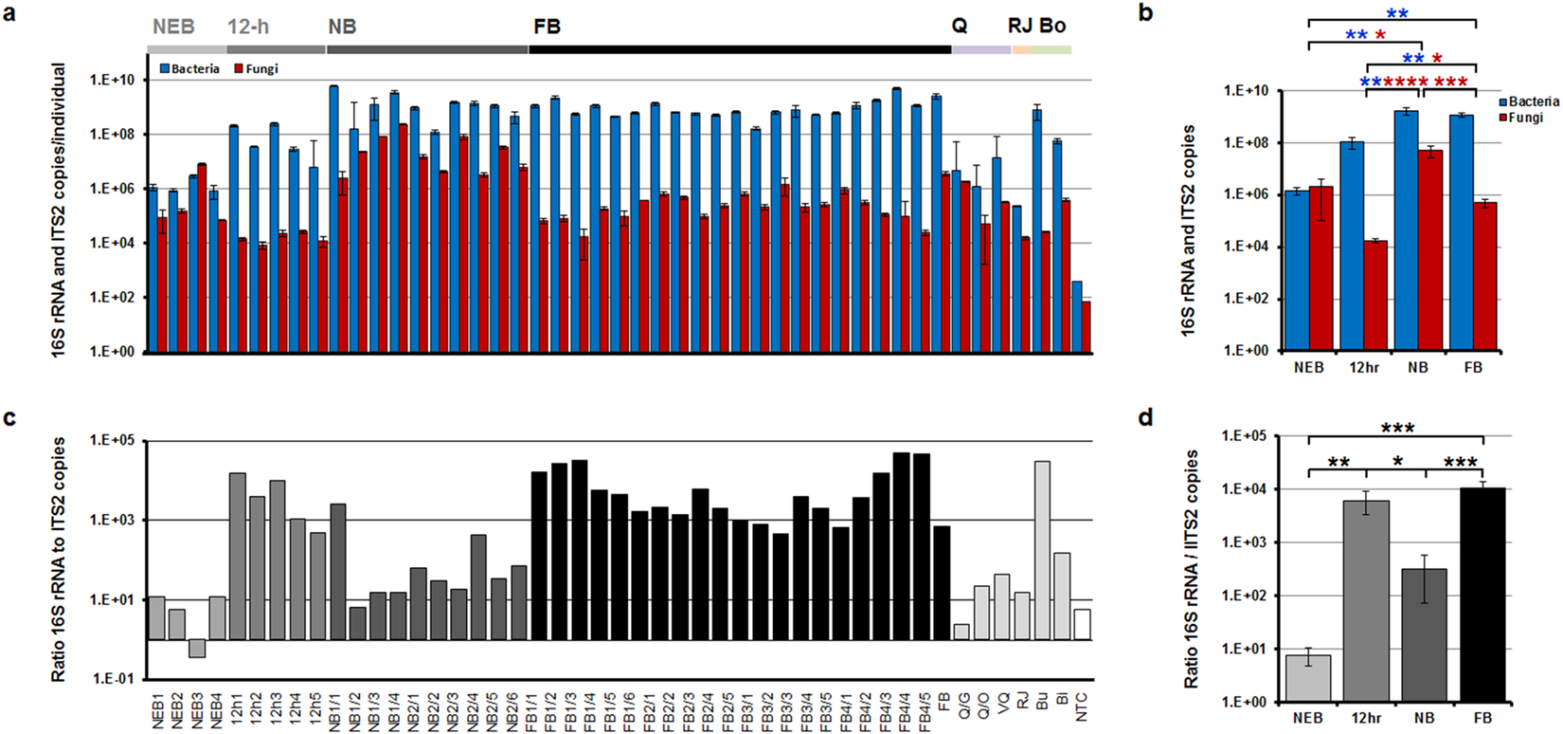
Microbial density associated with the social role of the honey bee. Comparison of copy number of *16S rRNA* and ITS2 region (**a**) of individuals and (**b**) of groups. The copy numbers were estimated by qPCR. (**c**) Normalization of the *16S rRNA* gene copies to ITS2 region copies (ratios) in individual samples. (**d**) The ratio of *16S rRNA* gene copies to ITS2 region copies analysed according to the host social role. NEB, newly-emerged bee; 12-h, 12-h-old bee; NB, nurse bee; FB, foraging bee; Q, queen; RJ, royal jelly; Bo,*Bombus*; NTC, no template control. Data are expressed as the mean ± s.e.m. An FDR adjusted p-value are from ANOVA after Kruskal-Wallis post-hoc test. Blue and red asterisks refer to statistically significant differences in the bacterial and fungal density, respectively. An FDR adjusted p-value; *P < 0.05, **P < 0.01, ***P < 0.001, and ****P < 0.0001.

To determine the differences in the gut microbial density associated with the social status of the host, we determined the absolute copy numbers of bacteria and fungi per individual gut using quantitative real-time polymerase chain reaction (PCR). The data revealed significant differences associated with the host social status (bacteria, *p* = 0.0002; fungi, *p* < 0.0001). The total detected *16S rRNA* gene copy numbers in *A*. *mellifera* workers (ca. 10^9,10^) were similar to previously reported data (Fig. 5a and b)^25^. The *16S rRNA* gene copy number in the newly-emerged bee was 1.45 × 10^6^ (±4.90 × 10^5^), and, after 12 h of adult eclosion, the bacterial gene copy number increased up to 1.05 × 10^8^ (±5.06 × 10^7^). The *16S rRNA* gene copies in the nurse bee and the foraging bee increased further, up to 1.69 × 10^9^ (±5.94 × 10^8^) and 1.17 × 10^9^ (±2.32 × 10^8^), respectively. Consequently, with age (from a newly-emerged bee to a nurse bee), *16S rRNA* gene copy numbers increased and were saturated at the nurse bee phase. The ITS2 copy numbers in the honey bee followed different patterns than *16S rRNA* gene copy numbers. Fungal ITS2 copy numbers in the honey bee, from a newly-emerged bee to the queen, were maintained at low level (below 10^6^). However, only in the nurse bee sample, the ITS2 copy numbers increased up to 4.95 ×10^7^ (±2.30 × 10^7^) (Fig. 5a and b), and resulted in a low ratio of *16S rRNA* to ITS2 copy numbers. The ratio of *16S* rRNA copy numbers to ITS2 copy numbers in newly-emerged bees (about 10^−0.5^–10^1.1^) and in the queen (about 10^0.3^–10^1.7^) was low (Fig. 5c and d). However, this was due to a relatively low *16S* rRNA gene copy number.

To examine the possible relationship between the abundance of specific gut bacteria and fungi, as affected by the host social status, we performed Spearman correlation analysis based on the taxa with significantly different relative abundances in LEfSe analysis. The correlation and statistical analyses revealed that several bacterial taxa were correlated with the specific populations of gut fungi (Supplementary Fig. S7). *Saccharomyces* was negatively correlated with both *Lactobacillus kunkeei* and *Lactobacillus* Firm-5 in newly-emerged bees. *Saccharomyces* was negatively correlated only with *L*. *kunkeei* in 12-h-old bees. *Saccharomyces* was negatively correlated with Firm-5 and positively correlated with *L*. *kunkeei* in foraging bees However, *Saccharomyces* was positively correlated with both *Lactobacillus* species in nurse bees. The relationships between *Lactobacillus* Firm-5 and *Saccharomyces*, and between *Snodgrassella* and *Saccharomyces*, were inversely correlated in the nurse and foraging bees. Therefore, we propose that the shift of the social status of the bee from nursing to foraging not only alters the gut microbial structure, but also changes the relationship between the members of gut microbiota.

## Discussion

We previously showed that the honey bee gut microbiota are distinct from other insects, and are constant regardless of geographical distribution^39^. The unique gut microbiota structure may change, depending on the monophyletic origin of the host^16^, social interaction^40^, and a unique diet (bee bread, honey and royal jelly)^14^. In the present study, the gut bacterial communities of the nurse and foraging bees were highly similar to those reported in previous studies, in contrast with those of 12-h-old bee and the queen^14^. Instead of resident bacteria, Enterobacteriaceae and Moraxellaceae were observed in the newly-emerged bee. Gammaproteobacteria are absent from the larval gut^25,41^, and Enterobacteriaceae are commonly found in the honey bee-related environment^15^. This reflects the notion that newly-emerged bees lose the gut microbiota that developed at the larval stage by shedding the gut lining during pupation; new gut microbiota are then acquired from the hive environment while the bee is chewing through the wax cap. Gut microbiota of a 12-h-old bee that had been exposed only to frame-stored components were dominated by *L*. *kunkeei*, likely originating from the bee bread or nectar^15^. In accordance with a previous study, the bacterial gut communities of the nurse and foraging bees have a similar composition (Fig. 2a), but the nurse bee gut community harboured a significantly larger proportion of Firm-5 and smaller proportion of *Snodgrassella* than the foraging bee (Fig. 2a, Supplementary Fig. S5, and Supplementary Table S2)^14^.

Sugar in the nectar is mostly composed of sucrose and its monosaccharides, glucose and fructose, and a previous study proposed that the honey is produced from nectar as a result of a metabolic transformation by both the worker bee and its gut microbiota^42^. Based on PICRUSt analysis, the carbohydrate (fructose and mannose, starch and sucrose) and lipid metabolism gene clusters were more abundant in the nurse bee gut microbiota; on the other hand, a diverse gene cluster that included several metabolism, cellular processes, and environmental information processing genes, was more abundant in the foraging bee gut microbiota (Fig. 4b). As the foraging bee consumes more nectar and honey (carbohydrates sources) than bee bread (protein source), the foraging bee gut microbiota is exposed to relatively fewer amino acids than the nurse bee microbiota. Nevertheless, the amino acid metabolism gene cluster was more abundant in the gut microbiota of foraging bees than in that of nurse bees (Fig. 4b); this result supports the notion that differences in the host diet are not directly reflected in the abundance of genes in the gut microbiota. The increased abundance of these gene clusters is linked to microbes that colonised the foraging bee gut to a greater extent than that of other bee groups (Fig. 2a). Whereas *Snodgrassella*, which showed a significant increase in foraging bees (Supplementary Table S2), possesses a biosynthetic pathway for 21 proteinogenic amino acids and vitamins^42,43^, ‘Firm-5’ (which was more abundant in nurse bees) possesses the biosynthetic pathways for only three proteinogenic amino acids^42^. This might contribute to differences in the abundance of gene clusters between the gut microbiota of nurse bees and foraging bees. The *Snodgrassella* genome possesses a complete gluconeogenesis pathway rather than major routes for energy production and carbon metabolism^42,43^. The *Bartonella* genus (of which Alpha-1 is a member) also obtains carbon and energy from catabolism of amino acids rather than from carbohydrates^44^. The relative abundance of Alpha-1 in foraging bees was higher than that in nurse bees, although the difference was not significant. *Bartonella apis*, isolated from the honey bee, was positive for urease activity^17^; urease genes and the gene encoding glutamine synthetase, a key enzyme for the recycling nitrogenous waste products, were identified in the *Bartonella apis* genome^45^. Taken together with previous findings, the PICRUSTs results support the notion that colonization by a suitable gut microbe reduces resource conflict and competition with the foraging bee host.

Gut microbiota from the non-reproductive queen and mated queen samples had similar structures but were different with respect to the dominant taxa. While the non-reproductive queen gut was dominated by Alpha-2.1 and Alpha-2.2, the mated queen gut was dominated by *Lactobacillus* Firm-5. Although the queen gut microbiota analyses indicate different dominant taxa depending on the study, Alpha-2.2 (which exists in low abundance in the worker gut) is commonly more abundant in the queen gut^14,46^. In a previous study, Alpha-2.2 (closely related to genera within the Acetobacteraceae family) was shown to largely colonise the royal jelly and royal jelly-related organs, hypopharyngeal glands, and crop of a nurse bee^18^, and the gut of an early larval instar (that receives only the royal jelly)^41^; this suggests that Alpha-2.2 is passed to the queen through oral trophallaxis of the royal jelly by a nurse bee.

The compositions of bacterial gut communities of the nurse and foraging bees were similar, whereas the compositions of fungal gut communities of the two groups were different (Fig. 2). *Saccharomyces* were dominant in the nurse bee gut, but their relative abundance was significantly decreased and shifted toward multiple fungal species in the foraging bee gut. The diverse fungal community of the foraging bee may be influenced by the external activity at outside of the hive of the host bee, frequently exposed to external environmental conditions. *Saccharomyces* colonising the nurse bee may be simply a reflection of what the bee is exposed to in the food. However, the abundance elevation of *Saccharomyces* in 12-h-old bee which consume bee bread like nurse bee, was not observed (Fig. 5). Taken together, the results suggest that *Saccharomyces* might colonize nurse bees. In previous studies, insect-associated yeasts, such as *Saccharomyces* and *Candida*, were reported to play a role in the digestion of substrates by secreted enzymes (such as *β*-glucosidases, xylases, and cellulases) and in detoxification of toxic plant metabolites in the insect host^47^. The nurse bee ingests mature pollen to synthesise the royal jelly, suggesting that *Saccharomyces* may provide a material for pollen degradation or assist in royal jelly maturation.

Interestingly, the gut and ovary of the queen were dominated by *Zygosaccharomyces*. *Zygosaccharomyces*, similarly to Alpha-2.2, were less abundant in the worker bee and flourished in the queen samples. We hypothesised that, if the nurse bee is crucial for the establishment of bacteria in the queen bee gut, the queen fungal community should also be influenced by the nurse bee. To assess mismatches in sequence assignment caused by low database coverage, representative sequences corresponding to *Zygosaccharomyces* were checked against the BLASTn NCBI database (June, 2016), with matches to *Zygosaccharomyces mellis* and *Zygosaccharomyces siamensis* isolated from honey. To date, several *Zygosaccharomyces* sp. have occasionally been found in the beehive environment^48^, but high abundance of *Zygosaccharomyces* in the queen gut has not been reported. Since the queen consumes the royal jelly, a mainly fructose- and glucose-containing acidic fluid^49^, and the queen’s gut microbiota is dominated by Alpha-2.2 and *Lactobacillus*^14,18,46^, the queen gut seems to contain acidic substances and metabolites, such as acetic acid and lactic acid. In contrast with *Saccharomyces*, acetic acid tolerance of *Zygosaccharomyces* is possibly associated with the capacity to metabolize and transport the acetic acid in the presence of glucose^50^. Alternatively, the environmental factor of the queen gut, such as pH, microbial competitor or co-operator, may allow colonisation by the *Zygosaccharomyces*, rather than *Saccharomyces* that dominates the gut of the nurse bee.

This study revealed that the honey bee gut microbiota undergo a significant shift in composition in parallel to the transition of the social status of the host and, that consequently, microbial community within the host shift to adapt to the gut environment. We were unable to verify whether that is because of outside activities of the foraging bee; the foraging bee showed the highest bacterial and fungal diversity, richness (Fig. 1 and Supplementary Table S1), and total bacterial numbers (Fig. 5), whereas the total fungal number in the honey bee gut was highest in the nurse bee (Fig. 5). A previous study reported a competition for sugar substrates between typical *S*. *cerevisiae* and *Lactobacillus* species, and yeast growth and metabolism are inhibited by acidic bacterial products, which induce yeast flocculation^51^; the Spearman correlation analysis, however, revealed a positive correlation between *Saccharomyces* and *Lactobacillus* Firm-5 only in the nurse bee gut (Supplementary Fig. S7). The high relative abundance of both *Saccharomyces* and *Lactobacillus* Firm-5, and high fungal density (regard as *Saccharomyces*) in the nurse bee gut suggested that negative relationship between *Saccharomyces* and *Lactobacillus* Firm-5 may be slackened by the interaction among the host and/or other gut microbes in this particular environment. Acidic metabolites produced by *Lactobacillus* are utilised by the host and other gut microbiota^42^; thereby, *Saccharomyces* might be able to colonise the honey bee gut as a permissive environment.

The current study is the in-depth report on the fungal gut community of the honey bee investigated by high-throughput sequencing, and delineates both the fungal and bacterial gut communities, and the correlation between the bacteria and fungi in the honey bee gut. In summary, the honey bee gut microbial community reflects the host diet, its social status and the relationship between the bacteria, fungi and host niches. Our findings have been corroborated by marker gene amplicon sequencing, PICRUSt, and quantitative real-time PCR, and the existing literature, but lack accurate functional analysis of the specific taxa in the host gut. Hence, further *in vivo* studies might be required to elucidate the complex relationship between the fungal and bacterial symbionts, and the host social status. Ultimately, furthering knowledge of the gut microbial community, and understanding the interactions between members of the gut microbiota and the physiology of their hosts might offer insight into honey bee health, and help protect the honey bee from potential agents of honey bee decline.

## Methods

### Sampling

The honey bees (*Apis mellifera*) were collected from a single healthy hive to minimise genetic background variation. In September, 2014, one capped brood-filled frame was moved from colony at an apiary located in Namyangjusi (37°66 N, 127°14 E) to the lab, and kept in an incubator under constant temperature and humidity conditions (34 ± 1 °C; 90 ± 5%) until adult eclosion. Newly-emerged bees were collected as soon as they emerged from capped cells, and 12-h-old bees were collected 12 h after eclosion. Five nurse bees were also collected, as well as one non-reproductive queen, and one naturally-mated queen, from the same colony. A commercial royal jelly sample was prepared from a colony of the same apiary where the brood-filled frame was obtained. After sampling, all samples were stored immediately at −80 °C. The *16S* rRNA seuqnces and gDNAs from part of the nurse bee sample (the designated ‘BKS’ sample), foraging bees and *Bombus*, previously analysed by the authors^41^, were re-used for the bacterial community analyses and PCR amplification of the fungal ITS2 region. These foraging bees were collected from four foraging fields using insect nets; nurse bee BKS samples were obtained from an beekeeper at an apiary located in Goesan-gun (36°48 N, 127°47 E)^39^. One non-reproductive queen, one naturally-mated queen, four newly-emerged bees, five 12-h-old bees, 10 nurse bees, 22 foraging bees, a royal jelly sample, and two *Bombus* sp. samples were used in this study.

### DNA extraction and preparation for pyrosequencing

Whole insect bodies were washed twice with ethanol; the guts were removed from the individual insects and total genomic DNA was then extracted (crop to hindgut). The royal jelly (0.5 g) and ovary of the mated queen were also included in microbial community analyses. Gut and ovary samples were homogenised in STES buffer [0.5 mL; composed of: SDS (1%), Tris-HCl (0.2 M), EDTA (10 mM), and NaCl (0.5 M)] by shaking with glass beads (0.5-mm diameter) for 5 min. Genomic DNA extraction was achieved by phenol-chloroform-isoamyl and ethanol precipitation methods^52^. Extracted genomic DNA samples were purified using QIAamp Fast DNA Stool Mini Kit (QIAGEN, Germany).

For PCR amplification of bacterial *16S rRNA* and the fungal ITS2 region, primer sets, 8F/338R and 58A2F/NLB4, respectively, containing sample-specific barcode and linker sequences, were used^39,53^. DNA-free samples were included to check for a potential contamination of the buffer and primer sets, and PCR amplification was repeated five times (technical replicates). The thermocycling conditions were as follows: 10 min denaturation at 95 °C; followed by 25 cycles of 95 °C for 30 s, 58 °C for 45 s and 72 °C for 1 min; with a final extension at 72 °C for 10 min. After verification of PCR amplicon sizes by 1% agarose gel electrophoresis, products that yielded no visible bands were re-amplified during five additional PCR cycles. PCR products were pooled, and the bacterial and fungal PCR bands of correct sizes (350–500 bp) were purified. Fungal ITS2 PCR was performed for most samples (all samples except samples J031 and *Bombus* DS044); PCR amplification was unsuccessful for six samples (BKS04, BGS02, BGS03, BGS04, non-reproductive queen and royal jelly). Equimolar amounts of PCR products were combined; subsequent 454 pyrosequencing was done by Macrogen (Seoul, Korea), using a GS FLX Titanium (Roche 454 Life Sciences). Bacterial *16S rRNA* gene sequences from BKS, the foraging bee and *Bombus* samples were from our previously published study^39^.

### Bacterial 16S rRNA gene sequence analysis

Sequences generated by pyrosequencing were processed with mothur v.1.35.0^54^. In total, 45,231 bacterial sequences were obtained, and low-quality sequences (such as sequences <250 bp, containing more than one ambiguous base, or sequences of the *16S rRNA* gene primers and barcodes) were removed. Of the remaining 41,259 reads, 6,175 unique sequences were used for further analysis to increase the analysis speed. The unique sequences were trimmed to include the V1-V2 region, based on secondary structural alignment^55^, to improve the accuracy of the analysis. To remove sequence noise generated by pyrosequencing errors and chimeric sequences formed during PCR amplification of the *16S rRNA* genes, ‘shhh.seqs’ and ‘pre.cluster’ scripts, and ‘chimera.slayer’ script, were used, respectively. Consequently, 4,153 unique sequences (40,369 sequences in total) were retained. OTUs were determined based on a 3% distance level. *Archromobacter* sequences (3,972) that represented a potential bacterial contamination of buffer solution^56^ and singleton sequences (764), 653 sequences corresponding to *Thermus spp*. originating from the royal jelly were removed.

Next, 640 OTUs (34,980 sequences in total) were submitted to Greengenes database-based classification (13_8_release) using ‘classify.seqs’ script with the k-nearest neighbour algorithm. To resolve OTU taxonomic assignments, representative OTU sequences corresponding to bacteria known to be highly conserved colonisers of the majority of honey bee guts were queried using NCBI blastn. To calculate Good’s coverage, and diversity indices, ‘summary.single’ script of mothur was used, and the results are presented in Supplementary Table S1.

### Fungal ITS2 sequence analysis

In total, 160,112 sequences were obtained after pyrosequencing. Low-quality sequences (45,491) were trimmed (parameters: minlength = 250, maxambig = 1, maxhomop = 12, bdiffs = 1, qwindowaverage = 25 and flip = T); next, pyrosequencing errors and chimeric sequences were determined using ‘shhh.seqs’ and ‘chimera.uchime’ scripts, respectively. Finally, 112,704 sequences (38,687 unique sequences) were retained and the sequences classified as ‘protist’ (48) or ‘unknown’ (4,948) in the UNITE ver. 7.0 (unite.ut.ee) database were then removed. The remaining 107,708 sequences were used for clustering determinations with CD-HIT 4.6.1 (www.cd-hit.org), with 0.97% sequence identity parameters. The clustering revealed 1,379 OTUs, based on 3% dissimilarity level; 484 singletons were then removed. Finally, 107,224 sequences (895 OTUs) were used for further analysis, fungal taxonomy classification, and alpha and beta diversity analyses. Taxonomy assignments were performed against the UNITE databases (ver. 7.0.). Three samples with insufficient reads, 1110HEB4 (18), BGS05 (38), and BSL04 (1), were excluded from beta diversity analysis.

### Beta diversity analysis and community comparison

To determine the relationships between the host social status and the respective microbial communities, similarities of the microbiota membership and structure were calculated using a metric based on OTU richness (Jaccard) or abundance (ThetaYC). Phylotype diversity was determined using ‘phylo.diversity’ script after a phylogenetic tree was constructed for representative OTUs using ‘clearcut’ script within mothur. The generated Phylogeny Inference Package-formatted distance matrix, based on Jaccard and ThetaYC values, was used for PCoA and NMDS to determine microbial community differences in honey bees classified according to their social status. To test for significant effects of the social status on gut microbial structure, molecular variance analysis (AMOVA) and analysis of similarity (ANOSIM) in the mothur package were used. Interaction networks for OTUs shared by the honey bee groups were visualised using Cytoscape v3.0.1^57^. In a network graph, each node connects host samples (coloured circles) and microbial OTUs (grey circles), and the connection between each sample and the OTUs is represented by weighted lines defined as the absolute number of each OTU in each sample. To examine the differences in the relative microbial lineage abundance between the hosts of social status, LEfSe^58^ was used in Galaxy application (http://huttenhower.sph.harvard.edu/galaxy). LEfSe was determined with Kruskal-Wallis tests (<0.05) and pairwise Wilcoxon test (<0.05). The LDA score threshold was 4.0 for bacteria and 3.0 for fungi. PICRUSt 1.0.0 (http://picrust.github.io/picrust) was used for a predictive functional genomic analysis of the honey bee bacteria based on the Greengene *16S rRNA* gene dataset. Significant differences in gene content between the hosts of social status were detected at LDA score >3.0.

### Quantitative real-time PCR analysis

The absolute gene copy numbers of bacterial *16S rRNA* and the fungal ITS2 in the honey bee gut were assessed using quantitative real-time PCR to compare bacterial and fungal cell numbers. Template DNAs were amplified in triplicate using primer pairs bac1055YF/bac1392R (bacteria) and 58A2F/NLB4 (fungi)^53^ using SYBR^®^ Premix Ex Taq™ II (TAKARA, Japan). PCR conditions were as follows: initial denaturation at 95 °C for 15 s; followed by 40 cycles of denaturation at 94 °C for 20 s, annealing at 58 °C for 30 s, and extension at 72 °C for 45 s; and final extension at 72 °C for 5 min. Amplification for melting curve analysis followed the reaction, with a 0.5 °C increase from 72 °C to 95 °C. Genomic DNA from *Escherichia coli* K12 and *Candida albicans* KCTC 7270 was amplified to construct standard curves for bacterial *16S* rRNA and fungal ITS2. Each PCR product was purified using a QIAquick PCR Purification kit (QIAGEN). Standard curves were created by plotting the threshold cycle versus purified PCR product concentrations as previously described^59^. Gene copy numbers under the range of detection of the standard curve were assigned at threshold of 1.0 × 10^3^ copies corresponding to the lower detection limit for the bacteria and fungi.

### Statistical analysis

Results are shown as the mean ± s.e.m., unless stated otherwise. Differences between samples were considered significant when *P* < 0.05 (*P < 0.05, **P < 0.01, ***P < 0.001 and ****P < 0.0001). All statistical analyses, heat map analyses, and calculations of Spearman correlation coefficients were performed using GraphPad Prism 7.0.3. Multiple comparisons were carried out with non-parametric ANOVA (Kruskal-Wallis test) corrected by controlling the False Discovery Rate of Benjamini and Hochberg. Differences in gene content between bacterial gut communities of nurse bee and foraging bee were carried out with unpaired t-test with Welch’s correction.

The possible correlation between the abundance of fungal members and bacterial members, as affected by host social status, was determined by Spearman’s r correlation analysis with two-tailed statistical significance. The colour and asterisk in each cell indicate the correlation coefficient, *r*, and significant correlations, respectively.

### Data availability

*16S rRNA* gene and ITS2 region sequences have been were deposited in NCBI Sequence Read Archive and the corresponding accession numbers are provided in Supplementary Table S1.

## Electronic supplementary material

Supplementary information
