# Supplementary material for: Social status shapes the bacterial and fungal gut communities of the honey bee
Source: Sci Rep. 2018 Jan 31;8:2019. doi: 10.1038/s41598-018-19860-7 (PMC5792453; doi:10.1038/s41598-018-19860-7)
Supplement: Supplementary file 1 — Supplementary information [file 41598_2018_19860_MOESM1_ESM.pdf]

Supplementary Information

Social status shapes the bacterial and fungal gut communities of the honey bee

Ji-Hyun Yun, Mi-Ja Jung, Pil Soo Kim and Jin-Woo Bae*

Department of Life and Nanopharmaceutical Sciences and Department of Biology, Kyung Hee University, Seoul 130-701, Korea

***Corresponding Author:**

Jin-Woo Bae

Department of Life and Nanopharmaceutical Sciences and Department of Biology, Kyung Hee University, Seoul 130-701, Korea

Tel: +82-2-961-2312

Fax: +82-2-961-9155

E-mail: baejw@khu.ac.kr

Supplementary Tables

Supplementary Table S1. Sample characteristics and alpha-diversity indices of the honey bee gut microbiota. The operational taxonomic units (OTUs) and diversity indices were calculated using mothur v.1.35.0. with a 3% distance level. s.e.m.; standard error of the mean.

**Groups**	**Sample**		**Bacteria**										**Fungi** (Accession: SRR5097664)							
			Accession (SRA)	Reads	Coverage	Sobs	Chao	Ace	Jackknife	Shannon	Simpson	Phylo-diversity	Reads	Coverage	Sobs	Chao	Ace	Jackknife	Shannon	Simpson
Newly	NEB1	1110HEB01	SRR5108095	109	0.899	22.00	31.17	34.28	33.00	1.68	0.42	2.34	6,081	0.999	29.00	32.50	36.25	37.00	0.95	0.45
emerged	NEB2	1110HEB02	SRR5108091	196	0.908	36.00	57.86	59.80	55.82	2.51	0.16	3.14	506	0.996	6.00	6.50	9.00	0.00	0.84	0.54
bee	NEB3	1110HEB03	SRR5108086	250	0.952	39.00	45.60	58.04	51.00	2.90	0.09	3.29	971	0.999	5.00	5.00	6.00	0.00	0.82	0.51
	NEB4	1110HEB04	SRR5108099	176	0.926	35.00	43.67	47.24	48.00	2.40	0.23	2.90	18	0.889	3.00	4.00	0.00	31.02	0.43	0.78
		Average		182.75	0.921	33.00	44.57	49.84	46.95	2.37	0.22	2.92	1,894	0.971	10.75	12.00	12.81	17.00	0.76	0.57
		s.e.m.		29.13	0.012	3.76	5.46	5.88	4.92	0.25	0.07	0.21	1,409	0.027	6.12	6.85	8.03	9.89	0.11	0.07
12-h-old	12h1	923HEB04	SRR5108090	1,090	0.989	22.00	38.50	37.92	40.19	0.53	0.79	0.28	1,297	0.992	29.00	42.75	94.44	41.69	2.19	0.16
bee	12h2	923HEB06	SRR5108094	918	0.986	23.00	49.00	65.73	58.81	0.62	0.77	1.40	684	0.987	13.00	49.00	228.28	66.30	0.93	0.46
	12h3	923HEB07	SRR5108085	1,293	0.989	24.00	46.75	52.99	49.52	0.46	0.84	0.80	1,844	0.990	34.00	72.25	107.36	76.44	1.15	0.42
	12h4	923HEB08	SRR5108097	1,674	0.991	33.00	48.00	73.07	48.38	0.65	0.77	1.54	824	0.988	45.00	50.63	55.02	55.00	2.46	0.17
	12h5	923HEB09	SRR5108092	1,147	0.987	26.00	43.50	80.51	42.17	0.45	0.85	0.40	1,408	0.991	48.00	54.00	59.47	60.00	2.48	0.14
		Average		1,224.40	0.988	25.60	45.15	62.04	47.81	0.54	0.81	0.88	1,211.40	0.990	33.80	53.73	108.91	59.89	1.84	0.27
		s.e.m.		127.40	0.001	1.97	1.90	7.55	3.27	0.04	0.02	0.26	209.10	0.001	6.26	4.98	31.47	5.79	0.33	0.07
Nurse	NB1_1	1121HNB1	SRR5108083	1,816	0.979	104.00	145.17	188.78	146.07	3.49	0.04	3.54	1,692	0.996	18.00	20.14	30.20	24.00	1.00	0.49
bee	NB1_2	1121HNB2	SRR5108089	1,677	0.976	105.00	165.00	152.91	200.52	3.14	0.08	3.14	867	0.999	6.00	6.00	7.74	0.00	0.80	0.56
	NB1_3	1121HNB4	SRR5108087	1,732	0.987	62.00	85.00	111.59	85.91	2.02	0.27	1.84	136	1.000	3.00	3.00	3.00	0.00	0.77	0.49
	NB1_4	1121HNB5	SRR5108093	1,527	0.979	100.00	120.67	155.49	132.00	3.15	0.08	3.14	2,244	0.999	7.00	10.00	0.00	73.74	0.47	0.78
	NB2_1	BKS01	SRR5097658	409	0.958	37.00	52.11	88.68	54.00	2.15	0.23	3.65	977	0.989	20.00	38.33	54.40	38.81	0.99	0.47
	NB2_2	BKS02	SRR5097643	525	0.971	41.00	52.67	56.71	56.00	2.43	0.14	3.21	4,401	0.997	40.00	55.60	49.68	80.79	1.00	0.47
	NB2_3	BKS03	SRR5097663	211	0.924	44.00	57.33	58.86	60.00	3.07	0.08	2.60	3,875	0.996	30.00	56.25	51.22	71.27	0.97	0.49
	NB2_4	BKS04	SRR5097654	578	0.972	54.00	64.91	66.73	70.00	2.71	0.13	4.05								
	NB2_5	BKS05	SRR5097629	426	0.972	31.00	53.00	42.41	62.65	1.99	0.23	2.55	1,211	0.985	32.00	70.25	103.28	71.11	1.25	0.41
	NB2_6	BKS06	SRR5108098	1,642	0.981	84.00	125.33	151.27	134.02	2.91	0.10	3.93	11,425	0.996	102.00	149.05	159.68	153.18	1.21	0.41
		Average		1,054.30	0.970	66.20	92.12	107.34	100.12	2.71	0.14	3.16	2,980.89	0.995	28.67	45.40	51.02	56.99	0.94	0.51
		s.e.m.		211.50	0.006	9.30	13.64	16.36	15.83	0.17	0.02	0.22	1,155.00	0.002	10.10	15.31	17.39	15.99	0.08	0.04
Foraging	FB1_1	BGS01	SRR5097666	753	0.956	75.00	110.20	114.27	111.46	2.89	0.10	5.66	3958	0.998	65.00	68.00	68.05	72.00	2.56	0.13
bee	FB1_2	BGS02	SRR5097656	374	0.965	41.00	52.14	52.08	54.00	2.54	0.15	4.27								
	FB1_3	BGS03	SRR5097661	559	0.948	76.00	112.91	103.44	120.20	3.20	0.08	5.63								
	FB1_4	BGS04	SRR5097651	601	0.957	71.00	96.00	97.81	98.45	3.03	0.10	4.99								
	FB1_5	BGS05	SRR5097633	331	0.931	45.00	87.17	122.83	94.30	2.59	0.13	3.20	38	0.947	5.00	6.00	7.36	0.00	0.93	0.52
	FB1_6	BGS08	SRR5108084	1,729	0.987	70.00	83.59	96.68	92.00	2.38	0.21	3.14	2,531	0.987	114.00	143.33	143.09	151.82	2.93	0.11
	FB2_1	BJA01	SRR5097638	357	0.933	67.00	80.80	113.40	91.00	3.36	0.06	4.13	1,121	0.974	85.00	103.45	111.52	114.00	2.28	0.23
	FB2_2	BJA02	SRR5097667	563	0.941	79.00	116.71	159.34	118.89	3.45	0.05	4.99	1,357	0.975	79.00	112.00	123.10	115.02	2.14	0.23
	FB2_3	BJA03	SRR5097655	618	0.951	67.00	98.07	140.77	98.45	2.79	0.12	5.28	1,399	0.979	119.00	133.03	144.42	149.00	3.08	0.13

	FB2_4	BJA04	SRR5097637	739	0.953	85.00	130.77	153.17	143.78	3.10	0.10	5.16	1,554	0.972	202.00	230.22	229.51	245.00	4.48	0.02
	FB2_5	BJA05	SRR5097636	502	0.948	63.00	84.67	96.14	89.00	2.71	0.14	4.24	2,802	0.984	158.00	187.12	201.15	203.00	3.39	0.07
	FB3_1	BSL01	SRR5097648	343	0.936	49.00	72.10	106.41	72.93	2.80	0.11	2.88	629	0.944	91.00	140.58	129.73	150.61	3.76	0.03
	FB3_2	BSL02	SRR5097632	266	0.940	32.00	47.00	76.94	48.00	2.21	0.18	3.42	4,445	0.985	271.00	304.50	325.47	339.00	4.08	0.03
	FB3_3	BSL03	SRR5097645	234	0.936	33.00	59.25	80.14	65.58	2.74	0.09	2.55	2,589	0.987	164.00	184.31	186.33	197.00	3.96	0.04
	FB3_4	BSL04	SRR5097646	433	0.947	57.00	107.60	81.61	124.16	3.00	0.09	4.23	1	0.000	1.00	1.00	0.00	2.00	0.00	0.00
	FB3_5	BSL05	SRR5097672	320	0.953	39.00	50.67	54.61	54.00	2.35	0.20	3.02	2,112	0.977	183.00	214.33	222.73	231.00	3.85	0.05
	FB4_1	BSS01	SRR5097665	272	0.963	30.00	37.50	40.04	40.00	2.33	0.17	0.24	929	0.967	113.00	128.50	139.25	144.00	3.62	0.06
	FB4_2	BSS02	SRR5097628	397	0.927	62.00	120.00	144.02	137.96	3.19	0.08	3.85	332	0.940	47.00	64.27	71.88	67.00	2.72	0.13
	FB4_3	BSS03	SRR5097671	334	0.961	48.00	55.09	58.10	61.00	3.02	0.08	4.31	250	0.952	29.00	36.33	54.82	41.00	2.03	0.25
	FB4_4	BSS04	SRR5097668	475	0.960	44.00	65.38	87.66	64.46	2.51	0.14	3.24	8,021	0.999	75.00	77.77	80.95	84.00	2.41	0.19
	FB4_5	BSS05	SRR5097649	357	0.922	55.00	102.25	143.13	127.28	2.62	0.18	3.83	5,484	0.998	42.00	53.25	51.12	179.82	1.27	0.40
	FB	J031	SRR5097650	420	0.967	39.00	61.75	54.40	65.22	2.61	0.13	2.47								
		Average		498.95	0.949	55.77	83.26	98.96	89.64	2.79	0.12	3.85	2,197.33	0.920	102.39	121.56	127.25	138.07	2.75	0.15
		s.e.m.		66.33	0.003	3.56	5.77	7.50	6.58	0.07	0.01	0.27	502.40	0.054	16.89	19.08	19.73	20.68	0.28	0.03
	Q/G	Mated queen	SRR5108100	1,427	0.996	13.00	16.75	19.67	19.00	0.20	0.94	0.85	1,826	0.996	27.00	34.00	31.71	34.00	1.28	0.40
	Q/O	Mated queen Ovary	SRR5108082	1,420	0.992	22.00	38.50	90.77	37.89	0.49	0.82	1.72	25,218	1.000	73.00	86.20	80.27	144.28	1.50	0.35
	VQ	Virgin queen	SRR5108096	1,904	0.996	24.00	26.63	31.54	31.00	0.92	0.53	1.03								
	RJ	Royal jelly	SRR5108088	589	0.966	56.00	79.75	75.40	82.08	2.74	0.12	4.05								
Bombus ussutensis	Bu	DS043	SRR5097669	618	1.000	10.00	10.00	10.00	10.00	1.36	0.31	0.95	167	0.958	20.00	24.20	26.36	27.00	1.84	0.27
Bombus ignitus	Bi	DS044	SRR5097652	649	0.998	18.00	18.00	18.30	0.00	1.72	0.26	1.40								
		Total Average		744.26	0.961	48.77	71.20	85.23	76.17	2.30	0.26	3.03	2,749.33	0.957	62.38	78.40	89.33	91.56	1.92	0.30
		s.e.m.		78.75	0.004	3.52	5.23	6.30	5.98	0.13	0.04	0.21	702.40	0.025	10.15	11.57	12.55	12.68	0.19	0.03

Supplementary Table S2. Statistical significance of the relative abundances for taxa depending on the worker bee social status. Each value indicates significant differences between each pair of groups. The mated queen gut and ovary, and virgin queen gut samples were considered the same group as the queen only in this analysis. NEB, new emerged bee; 12h, 12-hour-old bee; NB, nurse bee; FB, foraging bee. Each value indicates significant differences between each pair of groups. An FDR adjusted p-value are from Kruskal-Wallis test. N.S., not significant.

**Bacterial taxa**	**Groups**	**NEB**	**12-h**	**NB**	**FB**	**Fungal genus**	**Groups**	**NEB**	**12-h**	**NB**	**FB**
Lactobacillus Firm-4	12-h	n.s.				*Saccharomyces*	12-h	n.s.			
	NB	0.0065	0.0065				NB	n.s.	n.s.		
	FB	0.0108	0.0065	n.s.			FB	0.0011	n.s.	0.001	
	Queen	N.s.	n.s.	0.0108	0.0205		Queen	n.s.	n.s.	n.s.	n.s.
Lactobacillus Firm-5	12-h					*Zygosaccharomyces*	12-h	0.0162			
	NB	0.0001	<0.0001				NB	n.s.	0.0129		
	FB	0.035	0.0126	0.0064			FB	n.s.	n.s.	0.0409	
	Queen	n.s.	n.s.	n.s.	n.s.		Queen	0.0125	n.s.	0.0125	n.s.
Lactobacillus kunkeei	12-h	n.s.				*Yarrowia*	12-h	0.0312			
	NB	n.s.	0.0004				NB	n.s.	0.0312		
	FB	n.s.	<0.0001	n.s.			FB	n.s.	0.0149	n.s.	
	Queen	n.s.	0.021	n.s.	n.s.		Queen	n.s.	n.s.	n.s.	n.s.
Alpha-1	12-h	n.s.				*Coprinopsis*	12-h	n.s.			
	NB	0.0444	0.0337				NB	n.s.	n.s.		
	FB	0.0061	0.0039	n.s.			FB	n.s.	n.s.	0.0497	
	Queen	n.s.	n.s.	n.s.	0.0142		Queen	n.s.	n.s.	n.s.	n.s.
Alpha-2.1	12-h	n.s.				*Schizopora*	12-h	n.s.			
	NB	n.s.	n.s.				NB	n.s.	n.s.		
	FB	0.0057	0.0036	0.0245			FB	n.s.	0.0493	0.014	
	Queen	n.s.	n.s.	n.s.	n.s.		Queen	n.s.	n.s.	n.s.	n.s.
Alpha-2.2	12-h	n.s.				*Cystofilobasidium*	12-h	n.s.			
	NB	n.s.	n.s.				NB	n.s.	n.s.		
	FB	n.s.	n.s.	n.s.	n.s.		FB	n.s.	n.s.	0.0268	
	Queen	0.001	0.0008	0.0036	0.0007		Queen	n.s.	n.s.	n.s.	n.s.
Snodgrassella	12-h	n.s.				Unclassified	12-h	n.s.			
	NB	n.s.	n.s.			Agaricales	NB	n.s.	n.s.		
	FB	0.0002	<0.0001	0.0051			FB	0.0276	0.0194	0.0035	
	Queen	n.s.	n.s.	n.s.	0.0011		Queen	n.s.	n.s.	n.s.	n.s.
Gilliamella	12-h	n.s.				Unclassified	12-h	n.s.			
	NB	0.0151	0.0112			Pleosporales	NB	n.s.	n.s.		
	FB	0.0112	0.0091	n.s.			FB	0.0273	n.s.	0.0049	
	Queen	n.s.	n.s.	0.0245	0.0151		Queen	n.s.	n.s.	n.s.	n.s.
Other taxa	12-h	0.0003				Unclassified	12-h	n.s.			
	NB	0.0024	n.s.			Ascomycota	NB	n.s.	n.s.		
	FB	0.0445	0.0029	0.0445			FB	0.0048	n.s.	n.s.	
	Queen	0.0006	n.s.	n.s.	0.0079		Queen	n.s.	n.s.	n.s.	n.s.
						0.5% > taxa	12-h	n.s.			
							NB	n.s.	n.s.		
							FB	0.0053	n.s.	0.004	
							Queen	n.s.	n.s.	n.s.	n.s.

Supplementary Figures

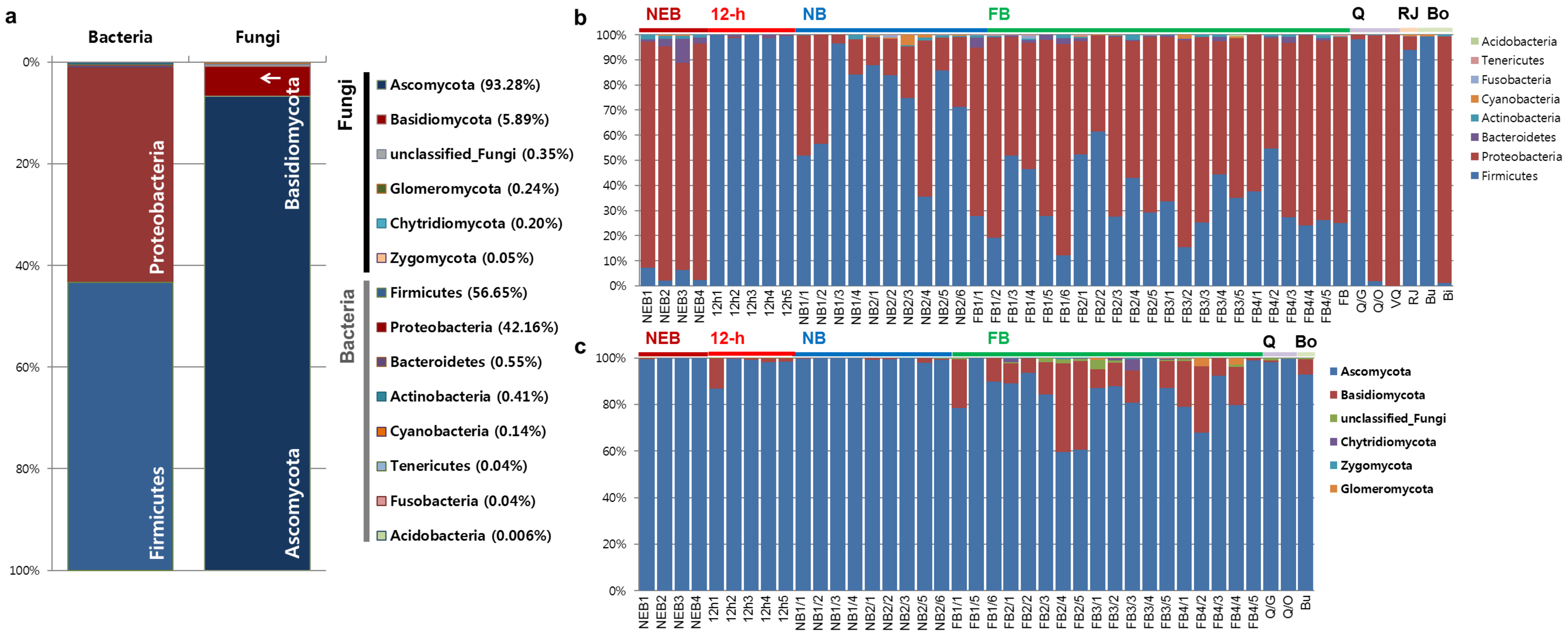

Supplementary Figure S1. Relative abundance and composition of the honey bee gut microbiota at the phylum level. (a) Total gut microbial community comprising eight bacterial phyla, five fungal phyla, and an unclassified fungal group. Changes in the relative abundance of the (b) bacterial and (c) fungal gut communities according to the social status of the host. NEB, newly-emerged bee; 12-h, 12-h-old bee; NB, nurse bee; FB, foraging bee; Q, queen; RJ, royal jelly; Bo,*Bombus*.

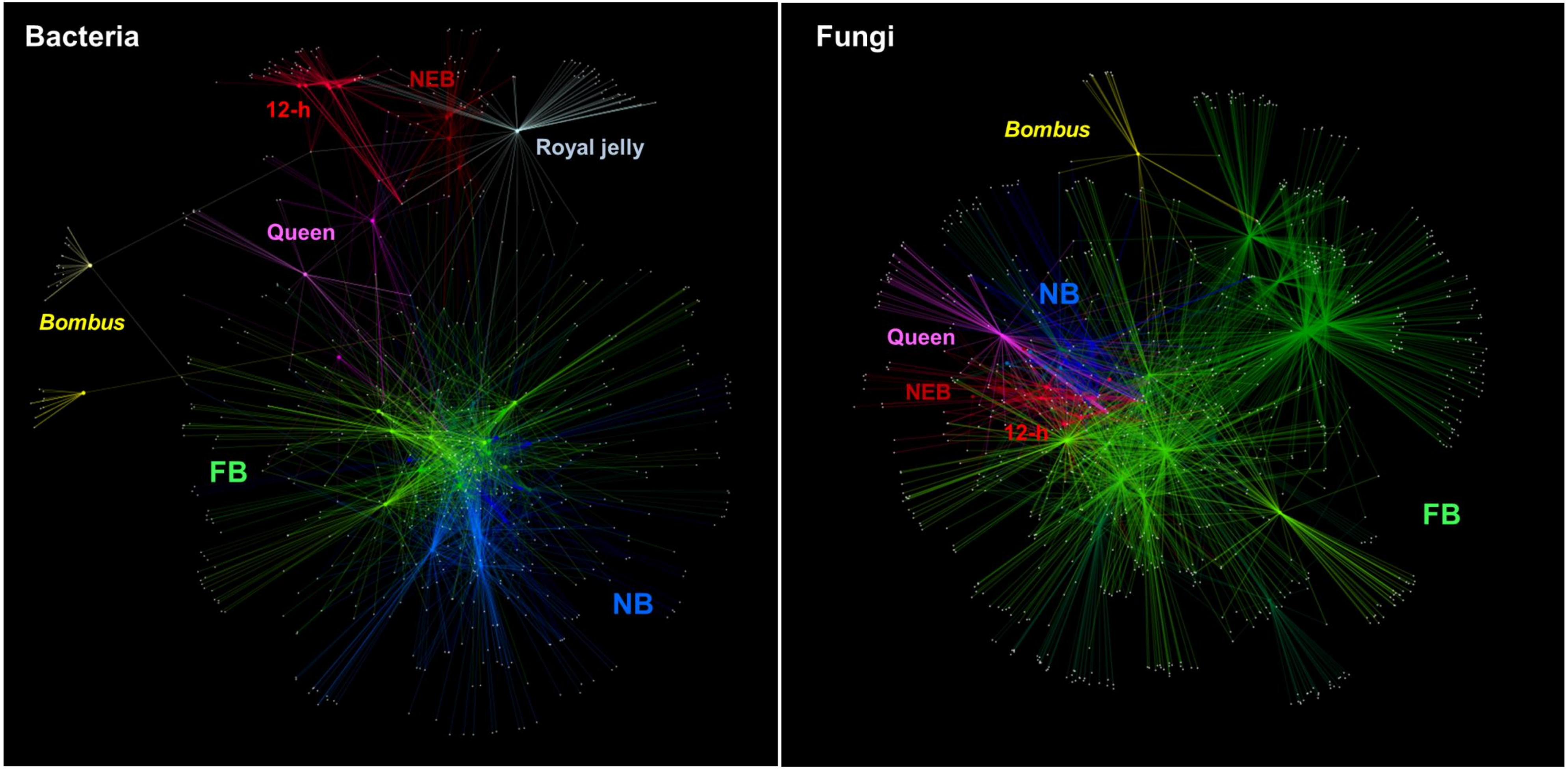

Supplementary Figure S2. Network analysis of the bacterial and fungal OTUs in the honey bee. Different social status of the host is designated by different colours. NEB, newly-emerged bee; 12-h , 12-h-old bee; NB, nurse bee; FB, foraging bee.

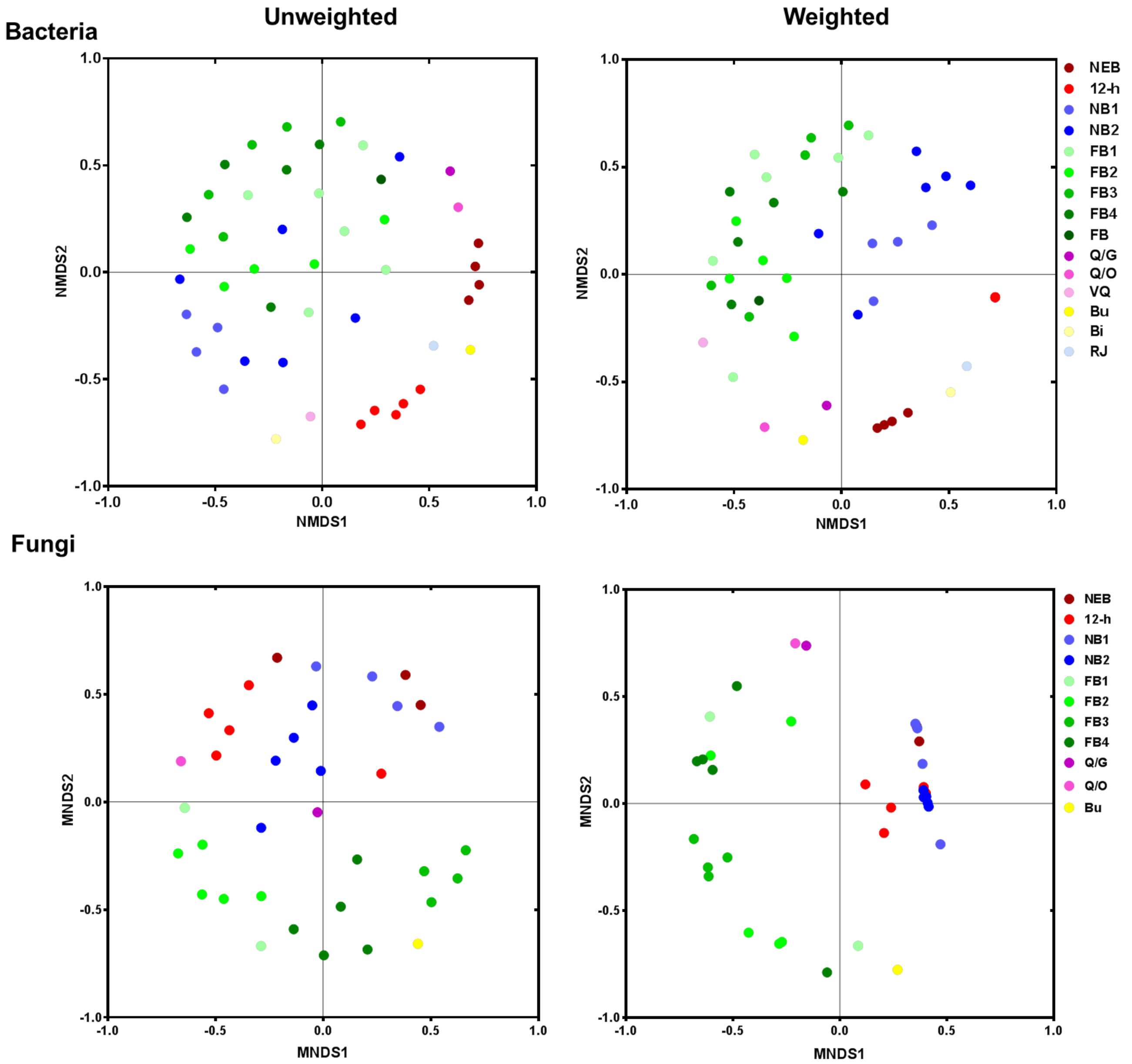

Supplementary Figure S3. Non-metric multidimensional scaling (NMDS) ordination plot based on the Jaccard (unweighted) and thetaYC (weighted) matrix of OTU-level bacterial (upper panel) and fungal gut communities (lower panel), and the honey bee social status. Different groups are represented by different colours. NEB, newly-emerged bee; 12-h, 12-h-old bee; NB, nurse bee; FB, foraging bee.

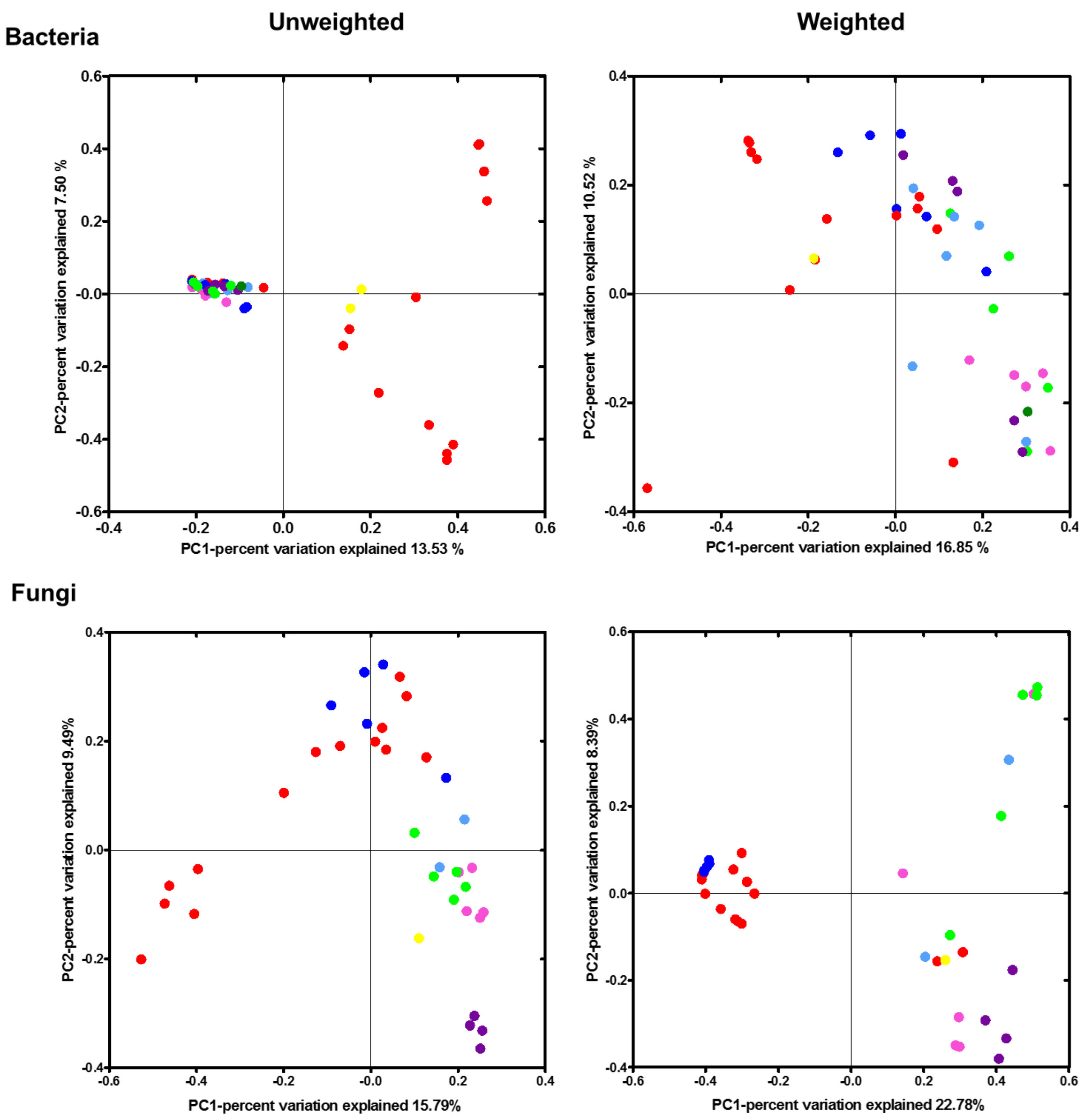

Supplementary Figure S4. PCoA plot based on the Jaccard (unweighted) and thetaYC (weighted) matrix of OTU-level bacterial (upper panel) and fungal gut communities (lower panel), and sampling sites. Different sampling sites are indicated by different colours.

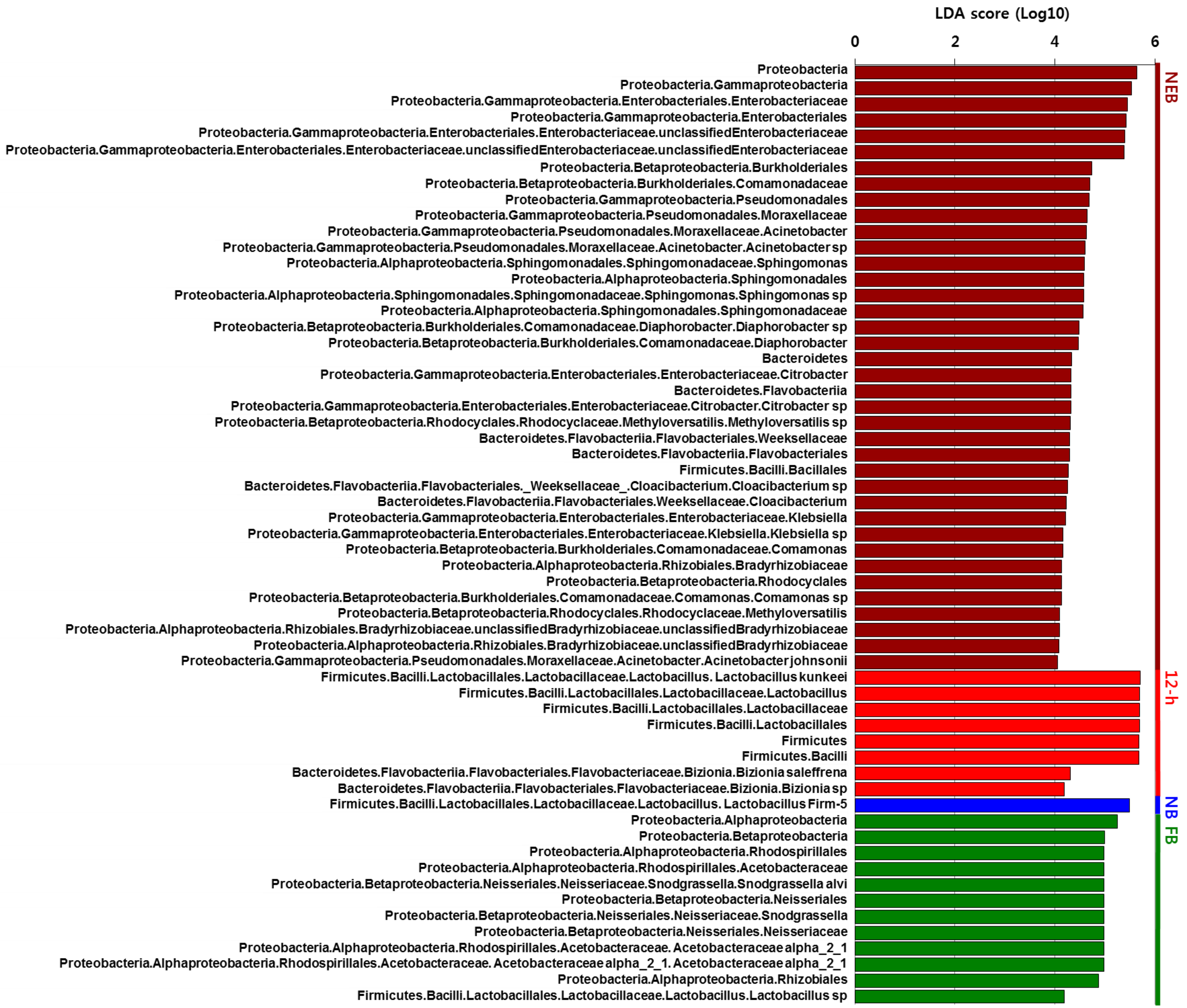

Supplementary Figure S5. Linear discriminant analysis (LDA) score histogram for differentially abundant bacterial lineages in gut samples from worker bees. Each colour represents bacterial clades over-represented in each group. LDA score threshold is 4.0. NEB, newly-emerged bee; 12-h, 12-h-old bee; NB, nurse bee; FB, foraging bee.

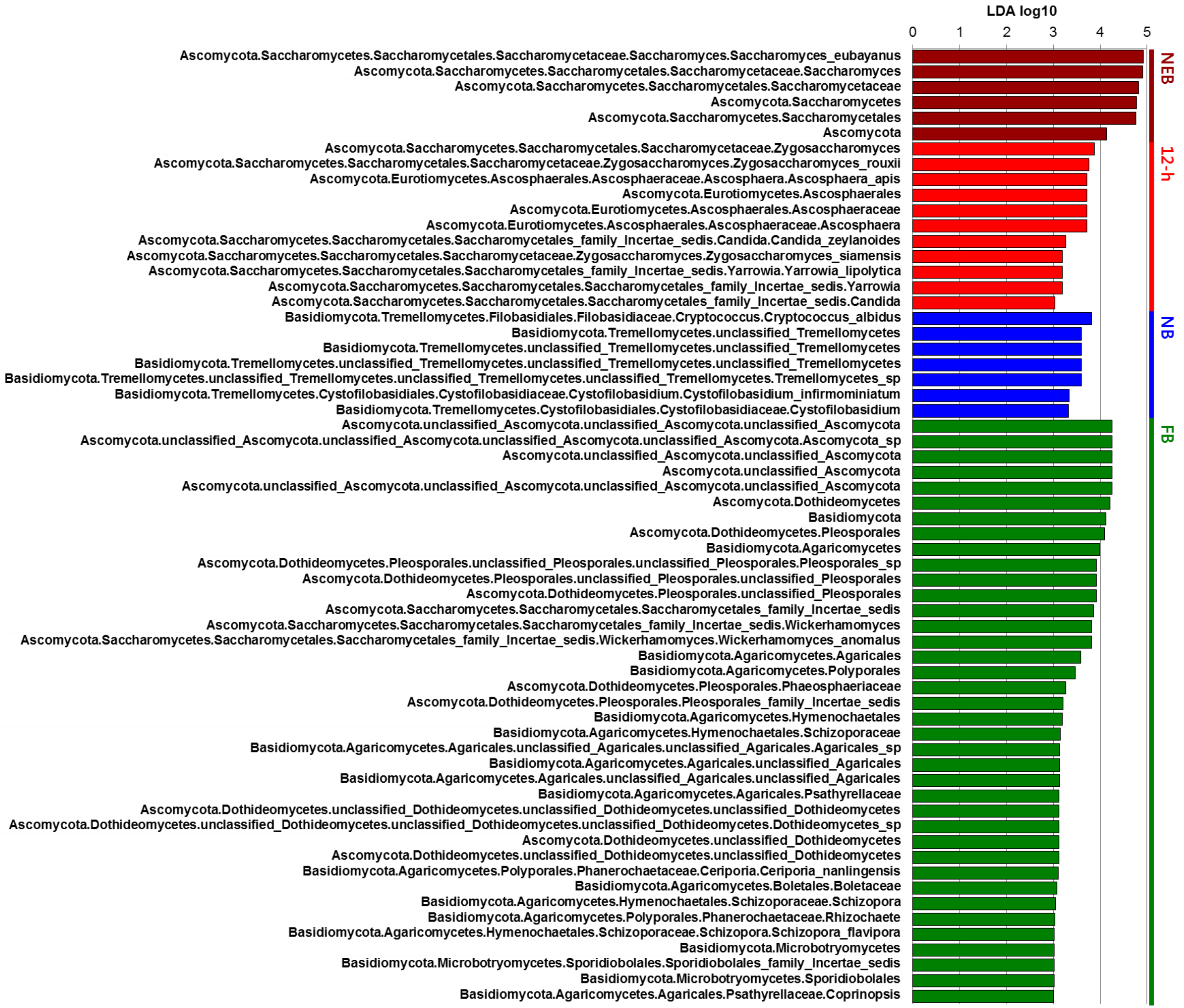

Supplementary Figure S6. LDA effect size analysis of the differences in the honey bee gut fungal abundances associated with the host social status. Output showing the effect size of 62 significantly enriched taxa in each group. LDA score cut-off is 3.0. NEB, newly-emerged bee; 12-h, 12-h-old bee; NB, nurse bee; FB, foraging bee.

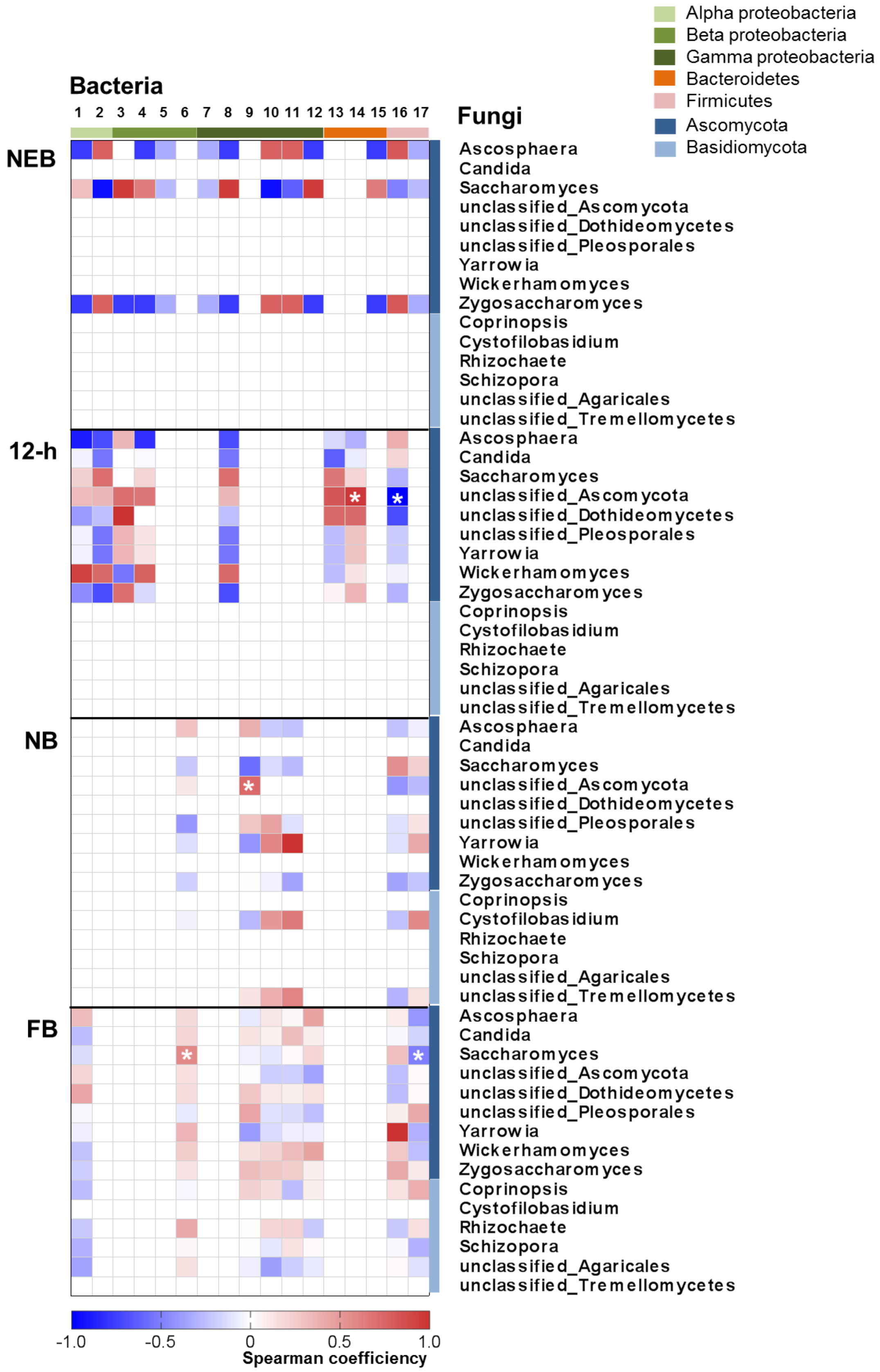

Supplementary Figure S7. Heat maps for Spearman correlation coefficients showing the association between the abundance of specific bacterial taxa and the abundance of specific fungal genera according to the honey bee social role. Each colour box represents a coefficient score. The asterisks in the grids demonstrate significant Spearman correlations as $^*P < 0.05$. NEB, newly-emerged bee; 12-h, 12-h-old bee; NB, nurse bee; FB, foraging bee. 1, *Sphingomonas* sp.; 2, unclassified Bradyrhizobiaceae; 3, *Comamonas* sp.; 4, *Diaphorobacter* sp.; 5, *Methyloversatilis* sp.; 6, *Snodgrassella alvi*; 7, *Acinetobacter johnsonii*; 8, *Acinetobacter* sp.; 9, Alpha-2.1; 10, unclassified Enterobacteriaceae; 11, *Citrobacter* sp.; 12, *Klebsiella* sp.;13, *Bizionia saleffrena*; 14, *Bizionia* sp.; 15, *Cloacibacterium* sp.; 16, *Lactobacillus kunkeei*; 17, *Lactobacillus* Firm-5.
